# A Brain Tumor Image Segmentation Method Based on Quantum Entanglement and Wormhole Behaved Particle Swarm Optimization

**DOI:** 10.3389/fmed.2022.794126

**Published:** 2022-05-10

**Authors:** Tianchi Zhang, Jing Zhang, Teng Xue, Mohammad Hasanur Rashid

**Affiliations:** ^1^School of Information Science and Engineering, Chongqing Jiaotong University, Chongqing, China; ^2^School of Information Science and Engineering, University of Jinan, Jinan, China; ^3^Shandong Provincial Key Laboratory of Network-Based Intelligent Computing, Jinan, China

**Keywords:** image segmentation, quantum entanglement, wormhole behavior, QPSO, QWPSO

## Abstract

**Purpose:**

Although classical techniques for image segmentation may work well for some images, they may perform poorly or not work at all for others. It often depends on the properties of the particular image segmentation task under study. The reliable segmentation of brain tumors in medical images represents a particularly challenging and essential task. For example, some brain tumors may exhibit complex so-called “bottle-neck” shapes which are essentially circles with long indistinct tapering tails, known as a “dual tail.” Such challenging conditions may not be readily segmented, particularly in the extended tail region or around the so-called “bottle-neck” area. In those cases, existing image segmentation techniques often fail to work well.

**Methods:**

Existing research on image segmentation using wormhole and entangle theory is first analyzed. Next, a random positioning search method that uses a quantum-behaved particle swarm optimization (QPSO) approach is improved by using a hyperbolic wormhole path measure for seeding and linking particles. Finally, our novel quantum and wormhole-behaved particle swarm optimization (QWPSO) is proposed.

**Results:**

Experimental results show that our QWPSO algorithm can better cluster complex “dual tail” regions into groupings with greater adaptability than conventional QPSO. Experimental work also improves operational efficiency and segmentation accuracy compared with current competing reference methods.

**Conclusion:**

Our QWPSO method appears extremely promising for isolating smeared/indistinct regions of complex shape typical of medical image segmentation tasks. The technique is especially advantageous for segmentation in the so-called “bottle-neck” and “dual tail”-shaped regions appearing in brain tumor images.

## Introduction

The accurate analysis of medical images, especially brain tumors, is essential in reducing clinical mortality rates. Brain tumors grow quickly and often appear as highly irregular and “complex shaped” in medical images. This characteristic tumor appearance is called a “dual tail sign” or “bottle-neck.” Usually, it occurs close to a meningioma, and the dual tail feature appears due to thickening, enhancement, and double distal tapering of the tumor in this area. Existing medical image segmentation methods often wholly ignore the smeared region or require long processing periods to obtain more accurate segmentation. However, precise medical image segmentation is essential in helping to better recognize and diagnose tumors. Thus, there is a pressing need for improved methods to help solve challenging tumor image segmentation problems. Many researchers believe that quantum theory offers a mysterious key that may help us interpret our future world (1–4). Significantly, the practical image segmentation method combines quantum theory with artificial algorithms (5–8), such as Quantum-behaved particle swarm optimization (QPSO). QPSO has been shown to perform well in clustering and image segmentation tasks involving complex object shapes (9). However, prior work with QPSO has not considered highly complex and irregular forms or indistinct smearing problems that are apparent in difficult medical image segmentation tasks.

Can we analyze the cause of the complex shape of brain tumors from the microscopic process and mechanism formation of brain tumor cells? What is the relationship between the complex shape of a brain tumor and the internal microscopic structure between the tumor cells?

In 2017, Maldacena and Susskind (10) reported that the fimbriae (finger-like threads appearing on bacteria) are visible as crooked tentacles, dragging DNA into the bacteria in a way that was somewhat analogous to the action of a wormhole between black holes. Particularly, it has been shown that a wormhole-like process exists in the synthesis of cells (11). Our previous research patent for invention (ZL200810209785.8) on the protein folding process proved that f(x) = acos(nx) + bsin(nx) could represent the oscillation of protein folding in a cell (12). The f(x) = acos(nx) + bsin(nx) is also a representation of a sin curve when proteins are in the folding process of forming a cell. It prompts the exciting question of whether there could be a wormhole effect amongst tumor cells.

Moreover, we might apply wormhole theory to improve QPSO for solving indistinct or highly complex 'bottle-neck,' smeared, or irregular shaped segmentation problems in medical images. To date, most wormhole physics has been applied in computing parallel connection problems or network attack prevention tasks and, to a limited extent, in ortholog prediction algorithms and gene clustering (13). However, the contribution and highlight of our research objective is to validate the application of wormhole theory to QPSO by proposing a novel method of quantum and wormhole-behaved particle swarm optimization (QWPSO) for complex medical image segmentation.

The rest of the study is organized as follows. In section Method, we first discuss the possibility of inducing wormhole behavior to achieve the complex shape in image segmentation. Then, we present the theory of wormhole path measurement and analyze the difference between wormhole path measurement and the Delta potential well measurement in the QPSO method. Finally, we put forward a novel segmentation method that we call QWPSO for complex shapes of brain tumors based on the wormhole path measurement. In section Results and Discussion, we apply the QWPSO algorithm to segment medical images, especially the complex shaped brain tumor images, and implement comparative experiments. Finally, some conclusions are given in the last section.

## Method

This section first discusses the possibility of similarity between wormhole behavior and the complex shape of brain tumor segmentation to primarily determine the tumor contour of “bottle-neck” and “dual-tailed.” Secondly, it analyzes QPSO algorithm and finally proposes the QWPSO algorithm to improve QPSO.

### The Possibility of Inducing From Wormhole Behavior to Brain Tumor Contour

The concept of “wormhole” was first proposed by Austrian physicist Ludwig Frum (14) in 1916 and was perfected by Einstein and Nathan Rosen (15) in 1935. Therefore, “wormhole” is also known as “Einstein-Rosen Bridge” (16). Worm-holes, commonly known as wormholes in space-time, is thought to be possibly curved shortcuts in the universe that allow objects to instantly travel through space and time. Figuratively speaking, a wormhole is a space tunnel connecting two distant spaces and times like a whirlpool in an ocean, ubiquitous but fleeting (17). These space-time vortices are caused by a combination of star rotation and gravity. Just as a whirl, it can make a part of a body of water closer to the bottom or make two parts of space that are relatively far apart become very close in an instant.

New research found that a wormhole's super strong magnetic field can keep it open by relying on a Phantom matter (18). Scientists believe that instead of a positive case, which produces energy, it also has a negative mass, sucking up all the energy around it. Because exotic matter has both positive energy and negative mass, it can create repulsive effects to prevent the wormhole from closing, thus stabilizing the “wormhole” energy field. In 2013, two distinguished theoretical physicists, Maldesina and Sarskander, explored the behavior of quantum entanglement in the macroscopic area. In their study, they boldly proposed the following: EPR = ER. EPR refers to quantum entanglement (19), and ER is short for wormhole (20). This puzzling formula links microscopic and macroscopic phenomena, pointing out that the exotic matter that stabilizes the wormhole energy field is quantum entanglement.

Wormhole features according to Maldesina and Sarskander (2013): physical space is by a space of two identical sheets, a particle being represented by a “bridge” connecting these sheets. The details are:

1. Wormholes are fragile and tiny (21).
2. The wormhole formation and wormhole stabilization process depends on a unique effect of exotic matter, which is the entangled state of quantum entanglement (22).
3. Changes in the magnetic field cause wormholes (23).
4. The shape of the wormhole is derived from the rotation of a baseline, and the baseline is hyperbolic. The embedded curved space is a hyperboloid (24).

**Figure 1:**
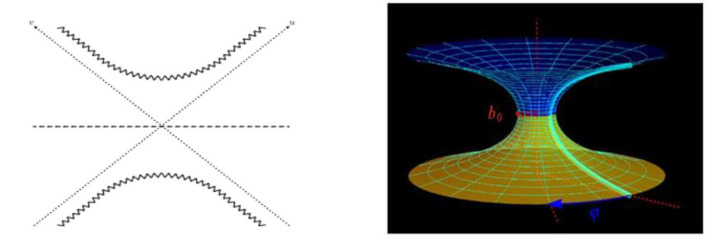
Wormhole hyperboloids. **(Left)** Lorentzian continuation of the Euclidean cigar. **(Right)** Schwarzschild metric.

Worm-holes can be described as the Lorentzian continuation of the Euclidean cigar. The Schwarzschild metric, shown in Figure 1, is the most famous wormhole model (25). It is a two-sided eternal black hole. The horizons are the diagonal dotted lines. The past and future singularities are the zigzag hyperbolas at the bottom and top (24).

#### Schwarzschild Metric Wormhole Model Equation

(1) $$\begin{array}{r} ds^{2}=-c^{2}dt^{2}+\frac{dr^{2}}{1-\frac{b_{0}^{2}}{r^{2}}}+r^{2}\left(d\theta^{2}+\sin^{2}\theta d\varphi^{2}\right) \end{array}$$

Where *c* is the speed of light, r is the radius of the throat part of the wormhole, θ is the zenith angle between positive z-axis, and φ is the azimuth angle between the positive X-axis in the spherical coordinate system. In the two-dimensional static spherically symmetric solution of a plane, Equation (1) can be simplified as:

(2) $$\begin{array}{r} ds^{2}=\frac{dr^{2}}{1-\frac{b_{0}^{2}}{r^{2}}}+r^{2}d\varphi^{2} \end{array}$$

Meanwhile, the equation of the embedded surface is:

(3) $$\begin{array}{r} z\left(r\right)=\pm b_{0}\ln\left[\frac{r}{b_{0}}+\sqrt{{\left(\frac{r}{b_{0}}\right)}^{2}-1}\right] \end{array}$$

where *b*_0_= 2GM (26), M is the object's mass, G is the universal gravitational constant, and r is the radius of the throat of the hyperbolic neck. Specifically, r is the distance of the curve represented by a radius line. At the same time, the wormhole's hyperbolic Equation (3) describes the spatial shape of the entire hyperboloid obtained by rotating numerous radius lines.

Roman Konoplya (27), a research associate at the People's Friendship University of Russia (RUDN) Institute for Gravity and Cosmology, proposed that the shape and mass of the wormhole can be calculated from the displacement value and the range of high-frequency gravitational waves. He first mathematically described the shape of a symmetrical wormhole based on its range of fluctuations. Then, using a quantum mechanical approximated the wormhole, we therefore simplified Equation (3) into Equation (4):

(4) $$\begin{array}{r} z\left(r\right)=\pm b_{0}\text{ln}\left(a\right) \end{array}$$

We used a hyperbolic disk to detail the equation for a hyperbola with angular momentum in all directions (4). The coefficient of *a* can be replaced by Δθ/2 and *b*_0_ can be replaced by 2/ζ. Hence, Equation (4) is written in detail as the following Equation (5):

(5) $$\begin{array}{r} x\left(r\right)=r+r^{{}^{\prime }}+\left(2/\zeta \right)\text{ln}\left(\Delta \theta /2\right) \end{array}$$

**Figure 2:**
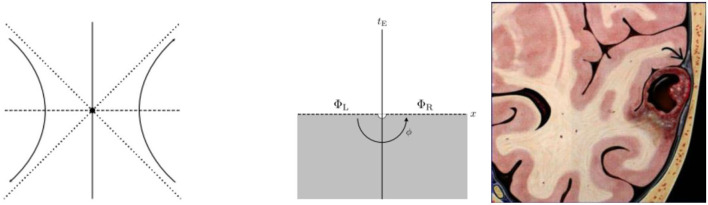
Wormhole equations and brain tumor in bottle-neck shape. **(Left)** Hyperbola of angular momentum. **(Middle)** Hyperbolic disk. **(Right)** Multitype xanthoma.

Some shapes of brain tumors look like 'bottle-necks' as hyperbolic shapes, such as the Multitype xanthoma shown in Figure 2. Is there any relationship between the shape of brain tumors and wormholes? Let us analyze in detail below.

#### The Cause of Brain Tumor

Mounting evidence specifically from long-term mobile phone use (cumulative exposure) shows that it can cause brain tumors, including glioma and acoustic neuroma, and appreciable long-term deficits in learning abilities and memory functions. Thus, it raises public concern and compels investigation (27–32). In Morgan's view (33), many results and several epidemiology studies are consistent with radiofrequency fields from which states that mobile phones can cause brain cancer. There are many causes to increase the risk of brain cancer, such as cumulative hours of use, long-time use, and radiated power. Saikhedkar's findings (34) indicate that extensive neurodegeneration on radio waves increased the unstable production of reactive oxygen. It is caused by the exhaustion of enzymatic and non-enzymatic antioxidants and increased lipid peroxidation. It indicates that radio waves increase the unstable production of reactive oxygen, causing extensive neurodegeneration in selective areas of CA1 (cornu ammonis 1), CA3 (cornu ammonis 3), DG (dentate gyrus), and the cerebral cortex. This extensive neuronal damage results in alterations in behavior related to memory and learning. Pronounced effects of electromagnetic fields may interfere with the results of laboratory tests on murine experimental models in veterinary or biomedical research (35). Electromagnetic radiations may result in chromosomal aberrations by either illegitimate recombination events or reduction of functionality of nonhomologous end-joining (36). An association with high-dose ionizing radiation and brain tumors has been observed in A-bomb studies, nuclear-test fallout data, therapeutic radiation for cancer and benign conditions, and occupational and environmental studies (37). Information is somewhat limited regarding the specific histologic type of tumor, particularly for increasing brain tumor risk. In 2015, the Swedish team and 13 other countries reported significant risks associated with gliomas from exposure to electromagnetic radiation, which was reclassified by the International Agency for Research on Cancer (IARC) from group 2B (probable) to Group 2A (highest level) (38, 39). Researchers found that mobile phone users had an eight-fold increased risk of brain tumors among people exposed to electromagnetic radiation in cities (40, 41).

#### The Similarities Between Wormholes and Brain Tumors

1. Both wormholes and brain tumors are caused by magnetic fields regardless of the super-strong magnetic field or electromagnetic radiation.
2. Their origin is the same because wormhole formation depends on quantum entanglement, while brain tumors are microscopic cells formed from mutated particles.
3. The formation processes of wormhole and brain tumors are all unstable as both are caused by exposure to magnetic fields.
4. Some brain tumors are the “bottle-neck” shapes that are the same as the hyperbolic shape of a wormhole

As for the cause, the initial formation process and the shape of brain tumors and wormholes are all similar. Hence, in the next section, we propose a wormhole behavior method to segment the “bottle-neck” shaped brain tumor.

### QPSO

The particle swarm optimization (PSO) method originally comes from a simulation of the social behavior of birds while flocking. However, PSO is not a global convergence-guaranteed algorithm. This is because at each iteration, the particles are restricted to a finite search space. Alternatively, the QPSO approach is one in which individual particles are assumed to have quantum behavior. QPSO is based on the quantum theory of a Delta potential well which offers a robust global searching ability (42, 43). Furthermore, the particles in QPSO can appear anywhere during the iterations, thus enhancing the population diversity.

In a Delta potential well, QPSO particles, in the process of optimization, move around the center area of the Delta potential toward the best position P for which the quantum potential *V*(*x*_*id*_) is expressed as *V*(*x*_*id*_) = −λδ(*x*_*id*_ − *p*_*id*_), where λ is weight, δ(*x*_*id*_ − *p*_*id*_) is the Dirac delta function, and *y*_*id*_ = *x*_*id*_ − *p*_*id*_. For the calculation of the particle's fitness values, we must know the exact particle position of *x*_*id*_. However, we only know the probability density function of *Q*(*y*_*id*_) from the quantum state of each particle *y*_*id*_ as shown below:

(6) $$\begin{array}{r} Q\left(y_{id}\right)={|\psi \left(y_{id}\right)|}^{2}=\frac{1}{L}e^{\frac{-2|y_{id}|}{L}} \end{array}$$

where *L* = *h*^2^/*mγ*, γ is the intensity of the potential well, *m* is the particle mass, and *h* is Planck's constant. As a given particle moves toward the potential well's center, ψ(*y*_*id*_) is the spin field operator, while the quantum state function *Q*(*y*_*id*_) represents the location of a particle probabilistically. To make the wave collapse to an actual state for each particle, we must use a method to estimate the position of the particles (44, 45). Employing the Monte Carlo random simulation (46), it is assumed that *s* is a lucky number within the range of (0, 1/*L*), that is:

(7) $$\begin{array}{r} s=\frac{1}{L}rand\left(0,1\right)=\frac{1}{L}u,\;and\;u=rand\left(0,1\right) \end{array}$$

Take Equation (6) into the random number Equation (7), $s=\frac{1}{L}e^{-\frac{2|y_{id}|}{L}},\;and\;u=e^{-\frac{2|y_{id}|}{L}}$. Consequently, $y_{id}=\pm \frac{L}{2}\text{ln}\left(1/u\right)$ and *y*_*id*_ = *x*_*id*_ − *p*_*id*_. Therefore, the estimated position of the particle *x*_*id*_ can be obtained by the following prototype:

(8) $$\begin{array}{r} \;X_{id}=P_{id}\pm \frac{L}{2}\ln\left(\frac{1}{\mu }\right) \end{array}$$

where *L* is the characteristic length of the potential well and μ indicates the random value between 0 and 1 that represents the arbitrary distance between particles in the quantum potential well. *P*_*id*_ is the best position of the particle.

Suppose *P* = (*P*_1_, *P*_2_, …, *P*_*M*_), then the particles coordinates of *P* is given by:

(9) $$\begin{array}{r} P=\left(\varphi_{1\;}\times P_{id}+\varphi_{2}\times P_{gd}\right)/\left(\varphi_{1}+\varphi_{2}\right) \end{array}$$

(10) $$\begin{array}{r} \text{Mbest}=\frac{\sum_{i=1}^{M}P\left(t\right)}{M} \end{array}$$

where φ_1_ = *rand*(0, 1), φ_2_ = *rand*(0, 1), *P*_*id*_ represents the *i*th components of the personal best position of the particle, and *P*_*gd*_ represents the global best position of the population. Mbest is the mean best position. The following iterative step is defined as the local best position of all particles on average and is calculated as follows:

If the random digital μ > 0.5,

(11) $$\begin{array}{r} \text{x}\left(\text{t}+1\right)=\text{P}-\text{α}\cdot |\text{Mbest}-\text{x}\left(\text{t}\right)|\cdot \text{ ln}\left(1/\text{μ}\right) \end{array}$$

If the random digital μ ≤ 0.5,

(12) $$\begin{array}{r} \text{x}\left(\text{t}+1\right)=\text{P}+\text{α}\cdot |\text{Mbest}-\text{x}\left(\text{t}\right)|\cdot \text{ ln}\left(1/\text{μ}\right) \end{array}$$

Where α is the expansion coefficient of the speed in controlling convergence, and it represents the maximum number of iterations.

### The QWPSO Method

Because particles in QPSO move around the central area of the Delta potential well, the existing QPSO approach, when applied to long-range searches such as when two regions are far apart, fails to segment well (47). However, there exists the notion of a wormhole in quantum theory. It offers an unusual correlation between particles, wherein actions performed on one particle immediately affect another reverse particle no matter how far apart the two particles are. We therefore propose a new quantum and QWPSO method, the details of which now follow. All nodes exist in a metric space, where distance abstracts to node similarities (48, 49). Hence, more similar nodes are closer in the area, and more alike or close nodes are more likely to be connected. Thus, particle optimization consists of links with the probability that decreases with the hidden distance. It gives two metric spaces between each pair of nodes: observable and hidden. Visible teams are joined with neighborhood nodes by entanglement (50, 51), while remote pairs can be expressed as a kind of wormhole.

Hence, we conclude that the features of the wormhole metric are as follows:

- All nodes exist in a metric space.
- The separation distance in this space represents one way of describing the similarity of the node. The more similar the nodes, the closer in the area they appear. Worm-holes link the other measure of similarity between nodes.
- The network consists of wormhole links. These exist with the probability that decreases with the hidden distance. Thus, more similar/close nodes are more likely to be connected.
- Worm-holes link long-distance nodes as a consequence of their negative curvature.
- A node forwards information to its neighbor closest to the destination in the wormhole space.
- Clustering is a consequence of the metric property of the wormhole spaces.

Worm-holes in Schwarzschild's solution form naturally in the cosmos, as it contains no matter and is merely full of curved space-time (52). Therefore, wormhole paths are asymptotically the shortest. However, many wormhole paths are successful depending on the image space geometry (51–59). Consequently, we put forward the measure that the wormhole is Hyperbolic in shape.

#### The Novel Wormhole Measure of Hyperbolic Path

Assuming a wormhole is a hyperbolic disc, we present the novel hyperbolic wormhole equation as of *N* = *ce*^*R*/2^, where R is the radius, *N* is the number of nodes in the network, and *c* controls its average degree. The node distribution of uniform angular density is ρ_θ_(θ) = 1/(2π), where the range of θ is from 0 to 57.32, namely, θ ≤ 360/2π. The node degree at a distance *r* from the disc center in an exponential radial density is ρ(*r*) = sinh *r*/(cosh *R* − 1) ≈ *e*^*r*−*R*^, and a simple approximation, ρ(*r*) ≈ (4*c*/π)*e*^(*R*−*r*)/2^ ≈ *e*^−ζ*r*/2^, connects each pair of nodes located at (r, q) and (r', q'), for which the connection probability is: *P* = *e*^ζ(*x*−*R*)/2^.

The wormhole measure of hyperbolic path *x* is as follows:

(13) $$\begin{array}{r} x=r+r^{{}^{\prime }}+\left(2/\zeta \right)\text{ln}\left(\Delta \theta /2\right) \end{array}$$

where the range of Δθ is: 0 < Δθ < 57.32 and ζ indicates the distance coefficient. When there is a wormhole between nodes, we modify the measure of QPSO as a wormhole path measure in a hyperbolic path of QWPSO.

#### QWPSO Method

The node probability distribution of the wormhole path measure ρ(*r*) is:

(14) $$\begin{array}{r} \rho \left(r\right)\approx e^{-\zeta r/2} \end{array}$$

The position of a particle in the wormhole path measure is:

(15) $$\begin{array}{r} X_{iw}=P_{id}\pm \left(2/\zeta \right)\text{ln}\left(\Delta \theta /2\right) \end{array}$$

*P*_*id*_ is the best position of the wormhole particle, and *x*(*t* + 1) represents the next step for the iteration variable wormhole particle which is defined as the local best position of all particles on average.

If the angle between nodes Δθ > 2, then

(16) $$\begin{array}{r} \text{x}\left(\text{t}+1\right)=\text{P}\left(\text{t}\right)-\left(2/\text{ζ}\right)\cdot |\text{Mbest}-\text{x}\left(\text{t}\right)|\cdot \text{ ln}\left(\Delta \theta /2\right) \end{array}$$

On the other hand, if the angle between nodes Δθ ≤ 2, then

(17) $$\begin{array}{r} \text{x}\left(\text{t}+1\right)=\text{P}\left(\text{t}\right)+\left(2/\text{ζ}\right)\cdot |\text{Mbest}-\text{x}\left(\text{t}\right)|•\text{ ln}\left(\Delta \theta /2\right). \end{array}$$

where Mbest is at the mean best position described as $\text{Mbest}=\frac{\overset{M}{\underset{i=1}{\sum }}P\left(t\right)}{M}\;$, *P*(*t*) represents the position of the particle *P*_*id*_ at time t, and M represents the number of particles.

#### The Difference Between QPSO and QWPSO

Our proposed QWPSO method is based on a measure of entanglement and wormhole theory. Using clustering, we firstly analyze and determine the connection type, i.e., is it entanglement or wormhole? If the connection is by trap, we find particles by a random link and cluster. If there is a wormhole connection between nodes, we employ our proposed wormhole measure, Equation (13), to find the particles and then cluster them. The main difference between QPSO and QWPSO is the coefficient α in equations (11), (12), and ζ in equations (16) and (17). ζ is related to distance, while α is related to speed. It means that while every step in QWPSO has a definite path, we know where to find a random process that finds the next particle in QPSO. Therefore, the efficiency in QWPSO is higher than QPSO due to the characteristic of a definite path exiting the wormhole. This is because ln(Δθ/2) in equations (16), (17) in QWPSO, and the range of Δθ is 0 < Δθ <57.32. Otherwise, in QPSO, the integer random value of μ in the function of *ln*(1/μ) is from 0 to 32767 depending on the computing power of a computer. Corresponding to the angular coordinate, the value range of μ in QPSO is from 0 to 360. Hence, 57.32 in 360 equals to 15.9%, the running time of QWPSO is only 15.9 % of the QPSO, and the efficiency of QWPSO is higher than that of QPSO. In addition, the critical difference between the existing QPSO approach and our QWPSO method is the definition of wormhole limitations. We conclude the three definitions for an existing wormhole as follows.

The three definitions for an existing wormhole:

(These limitations are more specific to image segmentation)

1. The number of nodes clustered by the wormhole is not less than two, i.e., there are at least two nodes as particles;
2. Node positions are not in the neighborhood, but their gray values are similar;
3. The similarity matches the wormhole measure.

If the cluster nodes meet the three limitations, the segmentation can be done by our proposed QWPSO method.

#### The Framework of the QWPSO Algorithm

**Figure 3:**
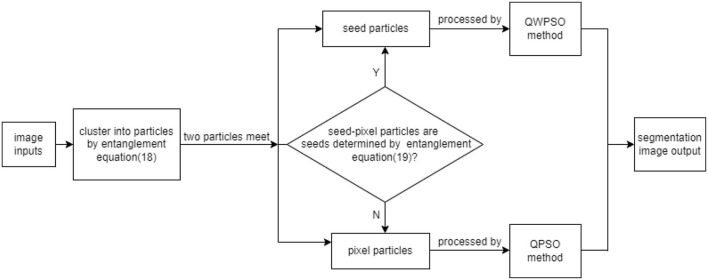
The framework of quantum and wormhole-behaved particle swarm optimization (QWPSO) algorithm.

As mentioned in previous sections, two distinguished theoretical physicists, Maldesina and Sarskander, explored the behavior of quantum entanglement in the macroscopic field. They boldly proposed the equation of “EPR=ER,” where EPR refers to quantum entanglement and E.R. is short for wormhole (6, 50, 60–64). The puzzling formula links microscopic and macroscopic phenomena and points out that the wormhole is caused by quantum entanglement. Inspired by this, this study presents the novel concept of seed and pixel particles. The seed particle is in quantum entanglement which exists a wormhole between each seed particle. In contrast, the pixel particle is opposite the seed particle, and there is no quantum entanglement and wormhole between the particles. Therefore, our proposed method of QWPSO consists of two sections. First, we cluster particles into seed and pixel particles. Secondly, we determine a wormhole between two seed particles by wormhole Equation (13) and segment the image using the QWPSO algorithm. Otherwise, if there are no seed particles, and therefore no wormholes between particles, the image segmentation is performed by QPSO. The detail of the QWPSO framework is shown in Figure 3.

1. Cluster particles into seed particles and pixel particles:

Two particles are found by a random process to determine if one particle is within the neighborhood range of the other particle. This is done by comparing the characteristics of the gray pixel value and position information between them. Assuming the two-particle positions, *x*(*i, j*) and *x*(*k, l*), their gray pixel values are *f*_*ij*_ and *f*_*kl*_. *TH*_*o*_ and *TH*_*f*_ are the threshold values of position variance and pixel gray value difference, where *f* represents the gray value difference of the two particles and Δ*d* represents the root mean square difference of the particles' position. Only when two particles satisfy the entanglement Equation (18) are they considered within one cluster as seed particles. Otherwise, they are pixel particles.

(18) $$\begin{array}{r} {\Delta f=|f}_{ij}-f_{kl}|\leq TH_{f}and\Delta d=\sqrt{{\left(i-k\right)}^{2}+{\left(j-l\right)}^{2}}\leq TH_{o} \end{array}$$

2. If a pixel particle encounters a seed particle:

If a pixel particle meets a seed particle, the gray seed value *f*_*kl*_ is replaced by the average gray value of the particles in the seed area, represented by $\bar{f}$. Only when two particles satisfy the entanglement Equation (19) are the two particles entangled together. They are then considered to be within one cluster as a new seed particle. The entangle equation is:

(19) $$\begin{array}{r} {\Delta f=|f}_{ij}-\bar{f}|\leq TH_{f}and\Delta d=\sqrt{{\left(i-k\right)}^{2}+{\left(j-l\right)}^{2}}\leq TH_{o} \end{array}$$

3. If two seeds meet:

Find a seed particle by Equation (13). If the two seed particles meet, and there exists a wormhole between them, then the entanglement equations (8), (11), and (12) are replaced by the measure of the wormhole equations (13), (16), and (17) respectively.

#### The QWPSO Algorithm

**Figure 4:**
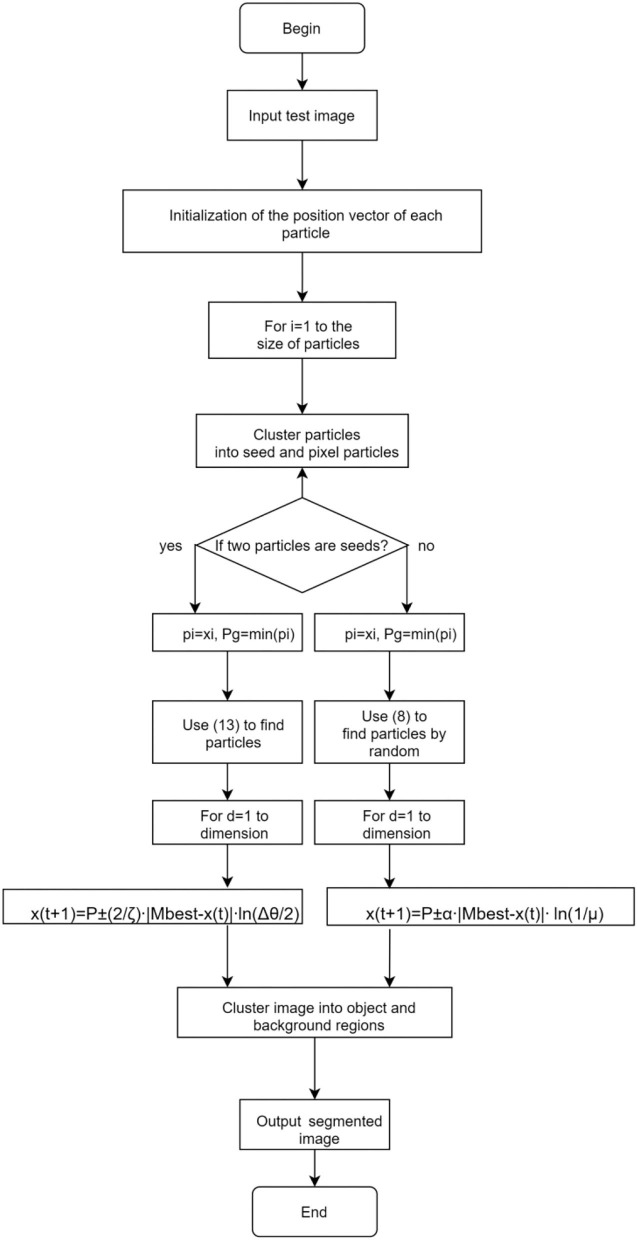
Flow chart of the QWPSO algorithm.

The process and flow chart of the QWPSO algorithm is shown in Figure 4. There are two sections in the algorithm. The left one is the seeds particle with wormhole path and the right one is pixel particles that have no wormhole path between them. According to the different paths, it will be processed with different equations.

The steps in conducting the QWPSO algorithm is listed below:

Step 1: Input the image and initialize the position vector for each particle.
Step 2: Cluster particles into seeds and pixels. In cases where two-pixel particles meet, check if one particle is within the neighborhood range of another particle by Equation (18), and then group them otherwise, go to step 5.
Step 3: In cases where pixel quantum particles meet a seed quantum particle, check whether any particle is within the neighborhood range of the seed particle using Equation (19). Then, group them. Otherwise, go to step 5.
Step 4: In cases where two seed particles meet, calculate their distance by Equation (13) and go to step 6.
Step 5: If the random digital μ > 0.5, then calculate according to Equation (11). Otherwise, calculate using Equation (12), and then cluster particles into foreground and background regions.
Step 6: If the angle between nodes Δθ >2, then calculate according to Equation (16). Otherwise, calculate using Equation (17), and then cluster particles into foreground and background regions.
Step 7: if all particles are clustered, output the segmented image and then exit. Otherwise, return to step 2.

#### The Contributions and Highlights of the QWPSO Algorithm

As for the complex so-called “bottle-neck” shapes in brain tumor image segmentation, to essentially solve the problem of 'dual tail' shape segmentation, we propose a novel method of QWPSO algorithm. The novelty, challenges, advantages, and limitations are as follows.

The novelty and challenge of the QWPSO algorithm:

1. The study of wormhole behavior comes from the microscopic process of DNA dragged into the bacteria. Between the tumor cells, we have sensed that fimbriae appeared as crooked tentacles to drag DNA into the bacteria, which is somewhat analogous to the action of a wormhole between black holes.
2. The study comes from the research of synthesis of cells as well. They prove that a wormhole-like process exists in the synthesis of cells.
3. The study origins from our previous research patent for invention (**ZL2008 1 0209785.8**) on the protein folding process proved that f(x) = acos(nx)+bsin(nx) could represent the oscillation of protein folding in a cell. The f(x) = acos(nx)+bsin(nx) is also a representation of a curve that looks like a worm-hole.
4. Based on the novel research of the microscopic structure of DNA into the bacteria, the wormhole-like process that existed in the synthesis of cells, and the protein curve folding process, we prompt an exciting and challenging research to discuss the relationship between the shape of the tumor and the shape of the wormhole.
5. This study's main contributions and highlights introduce a wormhole behavior method to improve QPSO into QWPSO. First, we proposed the novel concept of seeds and pixel particles. Thus, the QWPSO consists of two sections. Then, we present all the wormhole behavior equations, frames, and algorithms for QWPSO.
6. The difference or superiority of the proposed QWPSO method compared with the existing brain image segmentation methods mainly aims to solve the segmentation problem of special-shaped tumors, especially the shape of “bottle-neck” and “dual tail” based on the similar shape between the special shaped tumors and the shape of wormhole behavior.

The advantages of the QWPSO algorithm:

1. We propose the novel wormhole measure equation applied to the method of QWPSO. The wormhole measure is represented by the hyperbolic path, with angles describing the wormhole in all directions.
2. We propose the novel framework of the QWPSO algorithm with two sections. Firstly, the coarse clustering aims to achieve two groups of particles: seed particles have wormholes, and pixel particles do not. Secondly, and key to our method, is the refined clustering by quantum entanglement and the wormhole measure equation with seed particles.
3. The wormhole theory of a hyperbolic path in QWPSO is proposed instead of a random path as in QPSO. The running efficiency of QWPSO is higher than that of QPSO.
4. The QWPSO algorithm enables more accurately a segment in complex 'bottle-neck' and indistinct shapes, typical of trailing brain tumor images in cases where other segmentation algorithms often fail.

The limitations of the QWPSO algorithm:

1. The proposed QWPSO algorithm is designed for a brain tumor with a unique shape, but in a human tumor, there are various tissues and parts with such curved shapes, such as lung, liver, spleen, etc. Next, our study extends from brain tumors to image segmentation of other organs with curved shapes.
2. The proposed method of the QWPSO algorithm should be extended to the image segmentation of particular curved shape targets in other fields besides medical images. Therefore, we will study the application of this method and expand into more research areas in future studies.

## Results and Discussion

Magnetic resonance imaging and CT images are typically used to analyze medical brain images. In this section, we consider three tests for the two types of brain images. Test 1 included ten MRI brain images, including tumors of complex shape with long tails or bottle-neck contours. We wished to investigate whether our method is feasible and valuable in segmenting this challenging brain image, and determine the distance coefficient value of Δθ representing the angle between nodes. Test 2 aimed to test another important CT medical image beside the image of MRI for the comparative test to examine whether our proposed way is better than existing. There are four CT brain images for which we compare results with five current related reference methods. They are QPSO (65, 66), J. Sun cooperation quantum-behaved particle swarm optimization (SunCQPSO) (67), the Dynamic-context cooperation quantum-behaved particle swarm optimization algorithm (CCQPSO) (59), partitioned and cooperative quantum-behaved particle swarm optimization (SCQPSO) (45), and the improved quantum particle swarm optimization–intelligent fuzzy level set (IQPFLS) (8). We aimed to prove if our proposed QWPSO algorithm had better adaptability for object region shape, operational efficiency, and segmentation accuracy than QPSO and other typical competing reference methods. Test 3 is specifically for demonstrating the advantage of the proposed QWPSO method, the compared tests were implemented in 10 studies listed in references (8, 68–77), of which publication years were from 2018 to 2021.

### Test 1: MRI Brain Image Segmentation

Test 1 aimed to determine the distance coefficient value of Δθ in the proposed QWPSO approach. It consisted of 10 images, and they are from the benchmark datasets of brain tumor segmentation (BRATS) (78). Their names and tumor types are Glioma 1 and 2, Occipital, Ependymoma 1 and 2, Edema, Meningioma 1 and 2, Hematoma, and Tuberculoma. We aimed to test if our proposed method, QWPSO, could segment the tumor with complex object shapes called neck and tail features. Images of Ependymoma 1, Hematoma, Tuberculoma, and Ependymoma 2 include neck features, while the others all have tails features either long or short. From observation of the segmented images, shown in Table 1, it can be seen that the pictures with neck features, especially Ependymoma 1, Hematoma, and Tuberculoma as segmented by our QWPSO method, have a better and more accurate contour line than those segmented by using the QPSO method. The other images with tail features segmented by QWPSO also perform better than those segmented using the QPSO method.

**Table 1:** MRI brain image segmented by quantum-behaved particle swarm optimization (QPSO) and quantum and wormhole-behaved particle swarm optimization (QWPSO) methods.

**Method**	**Glioma 1**	**Occipital**	**Ependymoma 1**	**Glioma 2**	**Edema**
Original Image	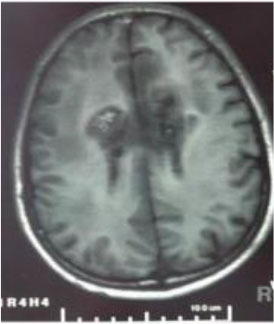	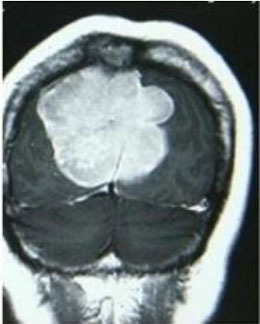	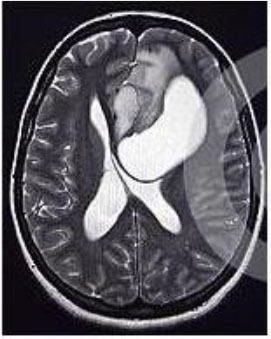	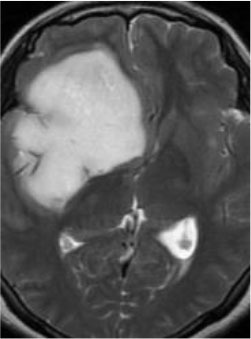	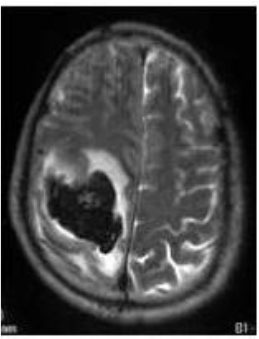
QPSO method	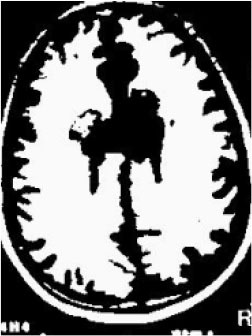	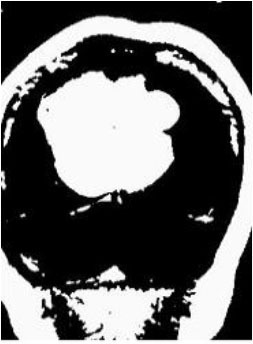	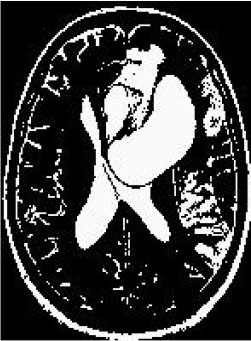	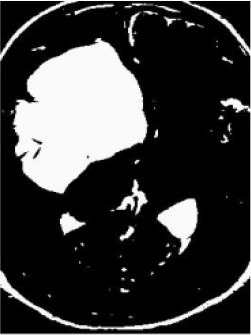	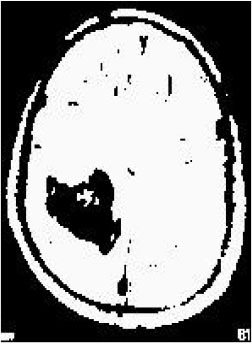
QWPSO method (Proposed in this paper)	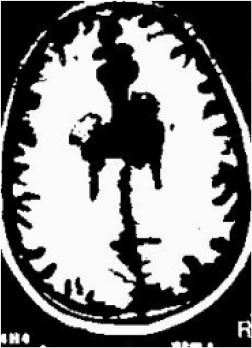	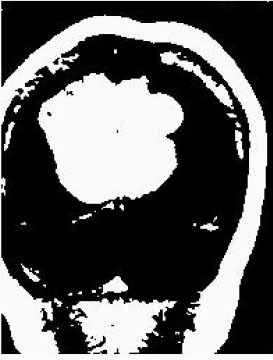	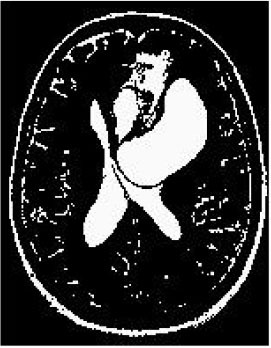	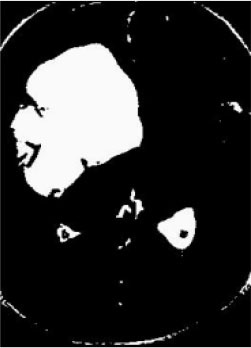	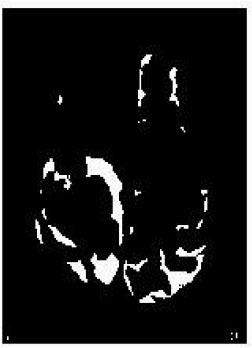
	Meningioma 1	Hematoma	Tuberculoma	Ependymoma 2	Meningioma 2
Original Image	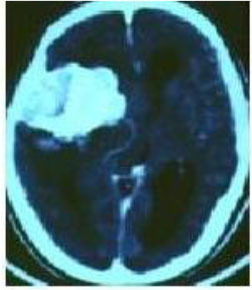	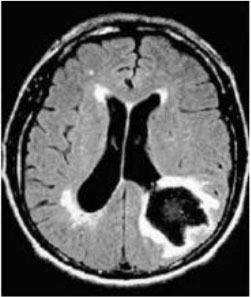	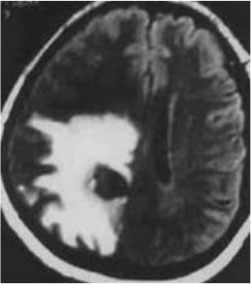	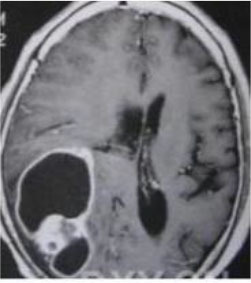	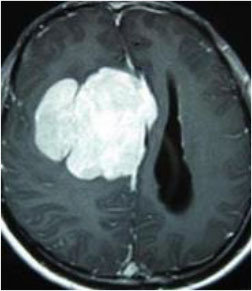
QPSO method	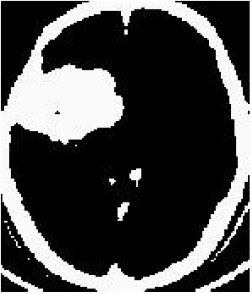	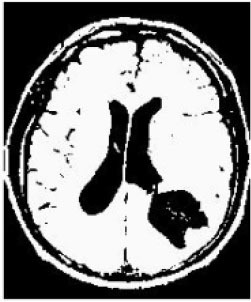	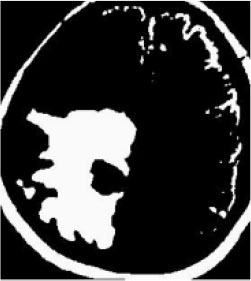	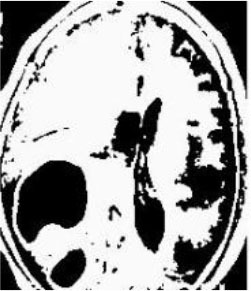	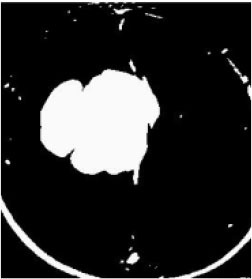
QWPSO method (Proposed in this paper)	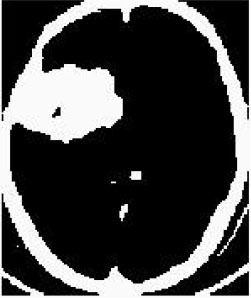	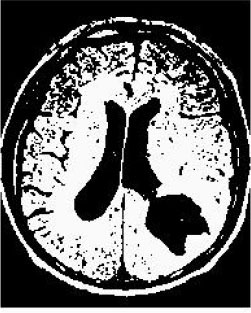	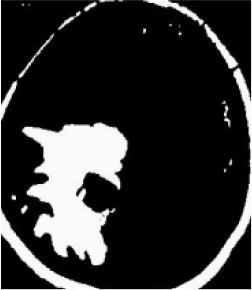	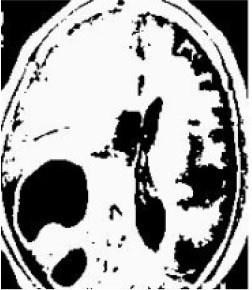	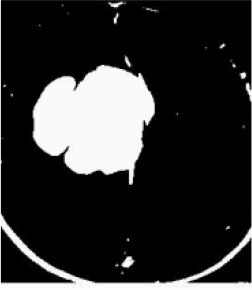

The quantitative evaluation parameters of the image segmentation process were Time (Running time), P (Precision), R (Recall), and F (F-measure). These were used to evaluate whether the method can achieve good results in image segmentation. The time parameters represented the running time to assess the algorithm's efficiency. P, R, and F were used to access and compare consistency, accuracy, and sensitivity, respectively. P is the fraction of retrieved relevant instances. It determines how beneficial the results are. The recall rate R is derived from our original sample, and it tells us how many positive examples in the sample were predicted to be correct. Finally, it was compared with the prediction. Therefore, P and R indicators are sometimes contradictory, so they need to be considered comprehensively by F. Specifically, P tells the accuracy, and F demonstrates the final and mixed evaluated results. The two parameters, P and F, are more critical among the P, R, and F parameters. The detailed evaluation parameters obtained from test 1 are shown in Table 2. The range of Δθ in the 10 brain images was 0.06 to 40.00. It is within the range we predicted and is less than 360/2π, which is within the scope of 0 to 57.32.

**Table 2:** Evaluation of parameters in test 1.

**Image**	**QWPSO evaluate parameters**					**QPSO evaluate parameters**			
	**Δθ/ rad/s**	**Time/s**	**P/%**	**R/%**	**F/%**	**Time/s**	**P/%**	**R/%**	**F/%**
Glioma 1	2.60	0.849	1.0000	0.9097	0.9527	0.863	0.9401	0.9090	0.9491
Occipital	2.00	0.865	1.0000	0.9903	0.9951	0.882	0.9996	0.9877	0.9936
Ependymoma 1	4.00	0.820	1.0000	0.9941	0.9970	0.840	0.9929	0.9988	0.9958
Glioma 2	40.00	0.829	0.9966	0.9868	0.9917	0.875	0.9741	1.0000	0.9869
Edema	1.60	0.823	0.9836	0.9941	0.9888	0.885	0.8344	1.0000	0.9097
Meningiom1	0.06	0.801	1.0000	0.9973	0.9986	0.950	0.9997	0.9888	0.9983
Hematoma	2.00	0.842	0.9995	1.0000	0.9967	0.976	0.9649	1.0000	0.9821
Tuberculoma	0.60	0.882	1.0000	0.9891	0.9945	0.922	0.9891	1.0000	0.9945
Ependymoma 2	2.00	1.144	0.9936	0.9256	0.9584	1.211	0.9048	1.0000	0.9500
Meningiom2	2.00	0.888	1.0000	0.9942	0.9971	1.000	1.000	0.9929	0.9964

The running time (Time) of our proposed method QWPSO was also less than the QPSO method. This is because our approach ran an angle Δθ within a changing range from in each step, while the QPSO ran a random value in every step. This meant that the changing rise in QWPSO had higher efficiency than the haphazard approach used in the QPSO method. Test 1 has also shown that the run time of QWPSO was less in the range of 84 to 98% than that of the QPSO. The evaluation parameters of P, R, and F, especially parameter P, for our QWPSO policy were optimal, i.e., they were all greater than the value obtained by QPSO. Moreover, the parameter R for QWPSO was better than that of QPSO. Furthermore, the parameter F was obtained from our proposed method, QWPSO, which was better than the method QPSO, except for the value 0.9945 for Tuberculoma, which is equal to both QPSO and QWPSO. In summary, for the essential evaluation parameters of Time, P, and F, our QWPSO method outperforms that which is obtained when using the QPSO method. This means that our process of QWPSO offers higher efficiency and greater accuracy than the QPSO method in the ten complex tumor-shaped medical image segmentation tasks.

### Test 2: CT Brain Image Segmentation

Test 2 aimed to test another important C.T. medical image beside the image from MRI. It explored the results compared with the latest improved QPSO methods: SunCQPSO, CCQPSO, SCQPSO, and the IQPFLS method. We considered four complex-shaped tumors that typically appear in CT medical tumor images, all of which included shapes with a long tail known as a 'dual tail' and the so-called 'bottle-neck' feature. The test CT images comprised CT201.86, CT201.136, CT201.29, and CT200.2, which were all taken from reference (78). The segmented images are shown in Table 3.

**Table 3:** Comparison segmentation test using CT brain images.

**Method**	**Image CT201.86**	**Image CT201.136**	**Image CT201.29**	**Image CT200.2**
Original image	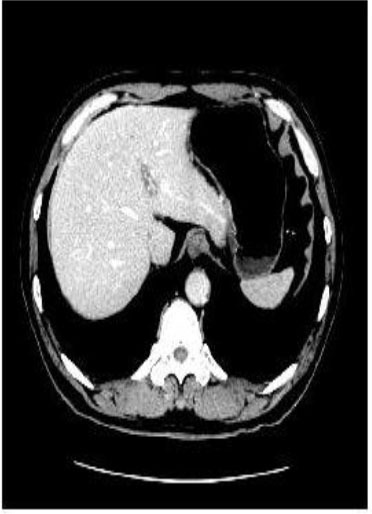	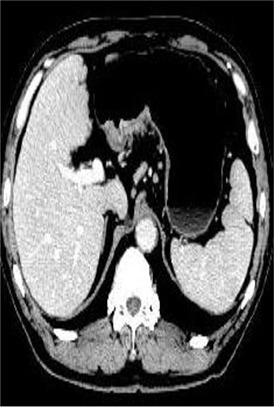	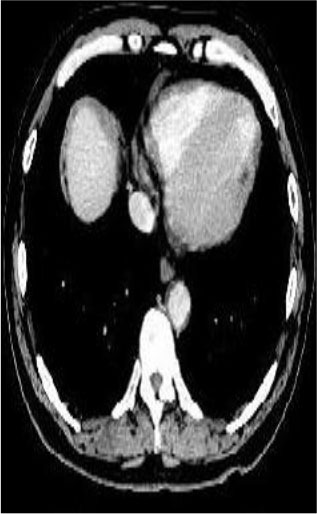	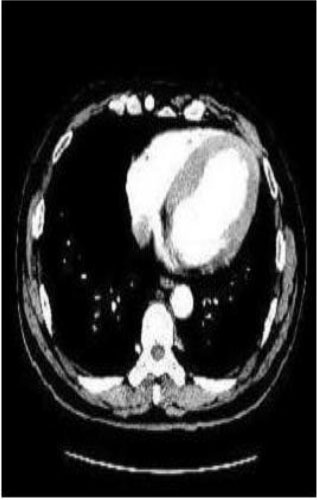
QPSO	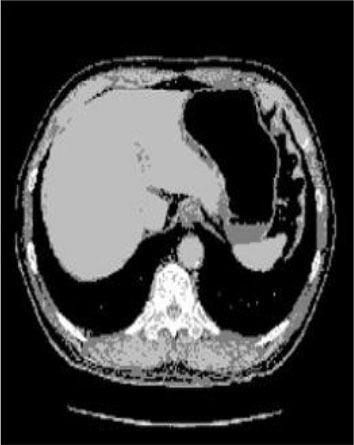	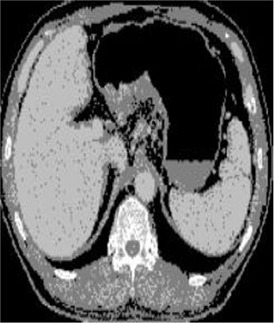	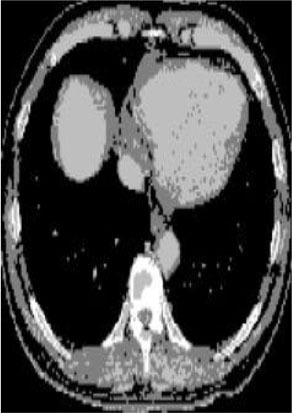	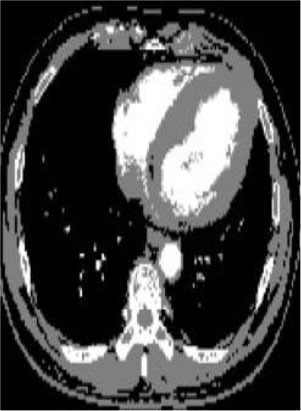
SunCQPSO	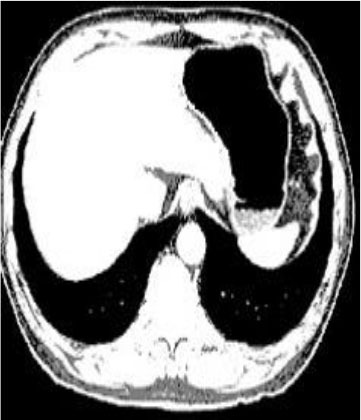	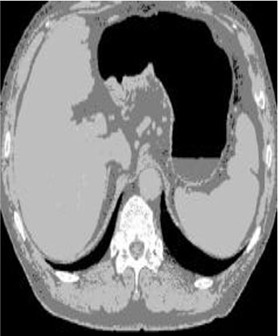	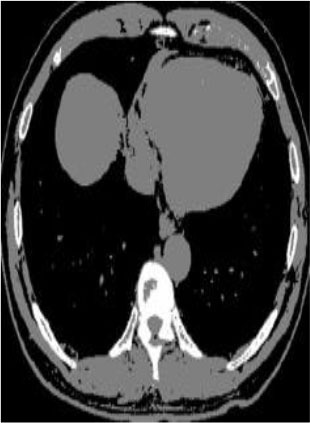	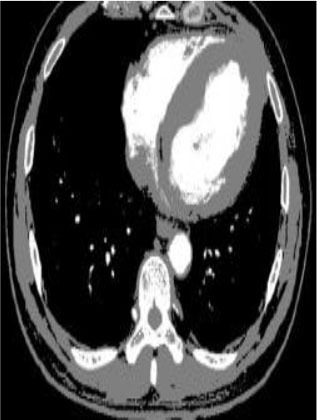
CCQPSO	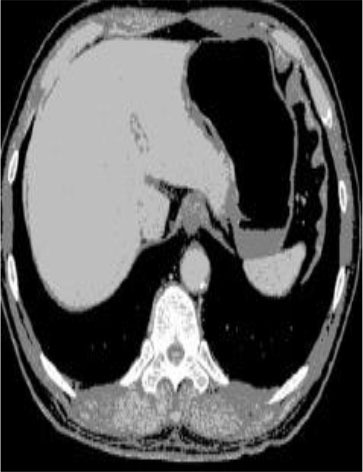	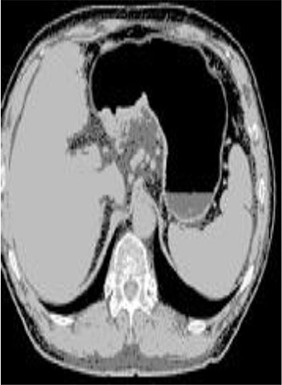	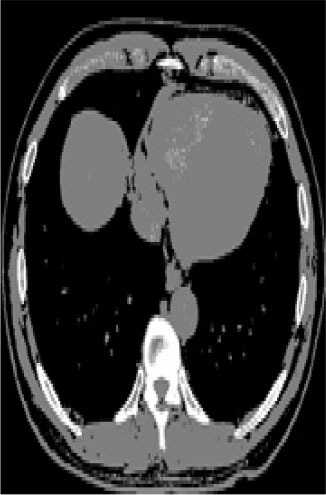	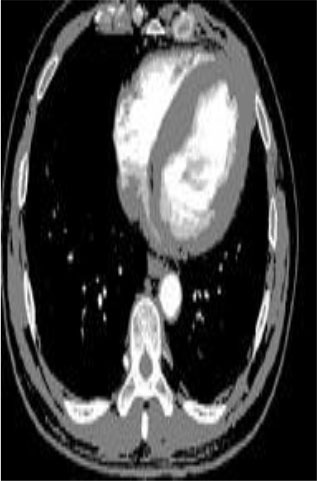
SCQPSO	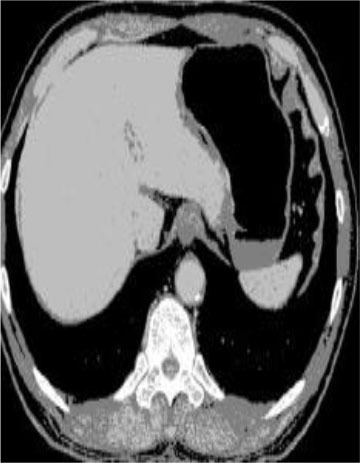	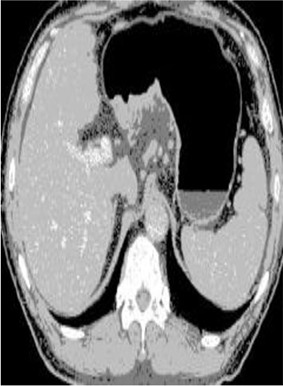	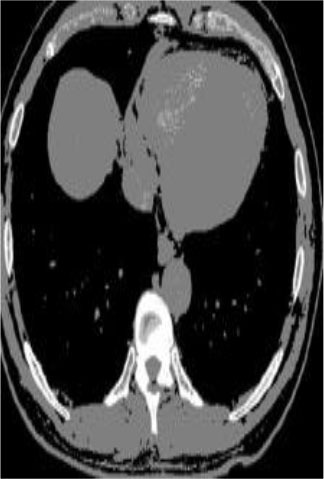	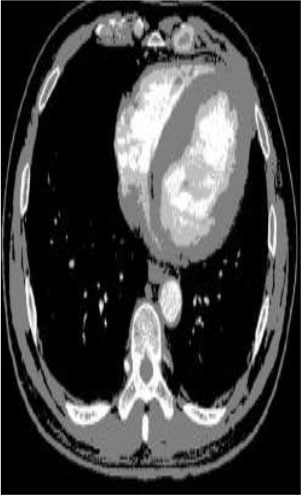
IQPFLS	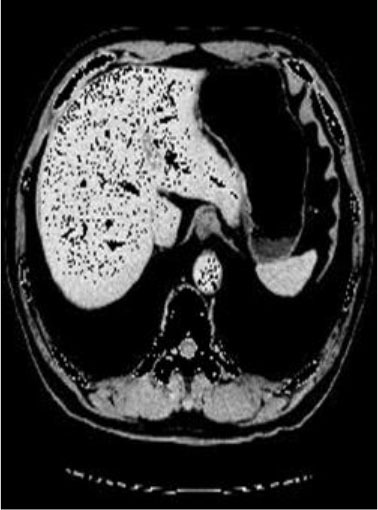	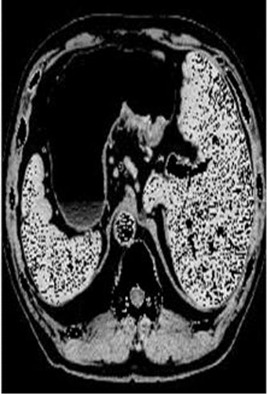	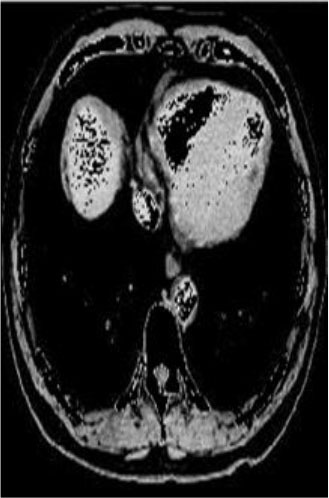	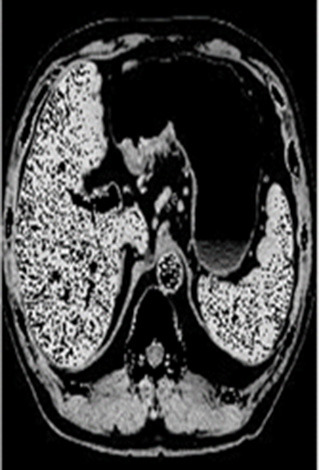
QWPSO (Our proposed method)	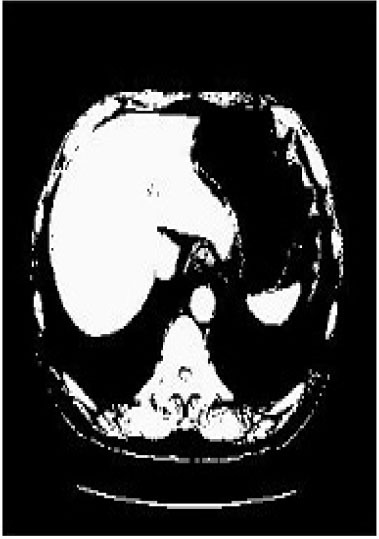	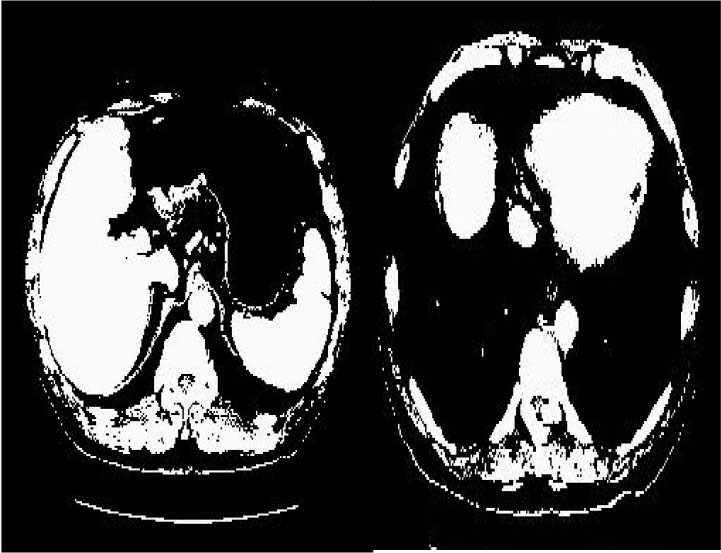	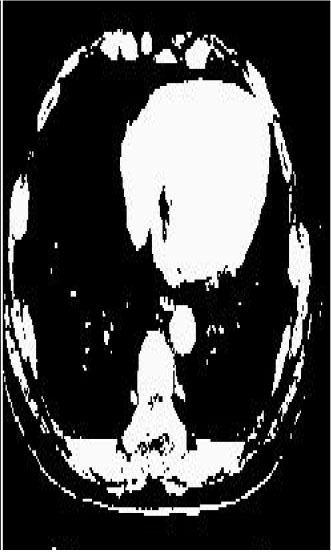	

In Table 3, it may be observed that the images segmented by our proposed method of QWPSO have a precise contour, especially at the region with the shape of long-tail known as the 'dual tail' and around the so-called 'bottle-neck' region. For example, in image CT201.86, there is a 'dual tail' in the object region. Thus, the trail is clear and distinct when segmented by our proposed method. However, the trail is comparatively fuzzy and unclear when segmented by competing methods like QPSO, SunCQPSO, CCQPSO, SCQPSO, and the IQPFLS method. Similar results were obtained for images CT201.29 and CT200.2. In the case of image CT201.136, there was a bottle-neck shape within the object region, for which our proposed method has achieved a distinct perfect bottle-neck curve in the object contour. On the other hand, neither a distinct 'bottle-neck nor angle was segmented by the other methods.

The evaluated parameters of P, R, and F are shown in Table 4. We use Δθ of 0.6 in our QWPSO method to segment the images. From Table 4, we can see that all the evaluation parameters (P, R, and F) show an improved performance for our QWPSO method compared with that obtained using QPSO, SunCQPSO, CCQPSO, SCQPSO, and IQPFLS. As for the mean values for R in our approach, QWPSO is all in the range of 0.7423 to 0.9990. Moreover, it is greater than the range of 0.3116 to 0.8876 obtained using the other five methods. The mean value R increased from 1.12 to 2.382 times. As for the value P, our QWPSO ways are all better than the compared methods except for our QWPSO and IQPFLS process in Image CT200.2 which had the same value of 0.7546. This is because there was no distinct 'bottle-neck' shape within the object region in Image CT200.2. The value of F is the combination of precision P and recall rate R. This reflects the total score of image segmentation. Our method QWPSO in the range of 0.7484 to 0.9995 is greater than the range of 0.3988 to 0.8171 obtained by the other five methods. The mean value F has increased 1.876 to 1.223 times. Namely, our proposed method QWPSO has significant advantages, especially in distinct 'bottle-neck' shape images. Furthermore, our approach's running time ranges from 0.810 to 0.900/s, which is less than needed for any of the other four methods.

**Table 4:** Evaluate parameters in test 2.

**Image name**	**Method name**	**Time/s**	**P/**	**R/%**	**F/%**
CT201.86	QPSO	0.932	0.6769	0.7985	0.7327
	SunCQPSO	1.000	0.6323	0.8876	0.7385
	CCQPSO	1.022	0.6822	0.8168	0.7435
	SCQPSO	1.120	0.6823	0.8164	0.7434
	IQPFLS	1.108	0.9476	0.7144	0.8146
	QWPSO	0.900	0.9996	0.9990	0.9993
CT201.136	QPSO	0.900	0.6078	0.5548	0.5801
	SunCQPSO	0.933	0.5670	0.6594	0.6097
	CCQPSO	0.912	0.5508	0.6220	0.5843
	SCQPSO	0.990	0.5734	0.6597	0.6135
	IQPFLS	1.102	0.9208	0.7344	0.8171
	QWPSO	0.856	0.9998	0.9993	0.9995
CT201.29	QPSO	0.912	0.6439	0.7887	0.7090
	SunCQPSO	1.000	0.5536	0.3116	0.3988
	CCQPSO	1.021	0.5657	0.3909	0.4624
	SCQPSO	1.020	0.6056	0.3355	0.4318
	IQPFLS	1.099	0.7571	0.6675	0.7094
	QWPSO	0.874	0.7944	0.7951	0.7948
CT200.2	QPSO	0.850	0.7216	0.6062	0.6589
	SunCQPSO	0.912	0.7186	0.6192	0.6652
	CCQPSO	0.923	0.7464	0.6583	0.6996
	SCQPSO	0.931	0.7654	0.6622	0.7101
	IQPFLS	1.111	0.7858	0.7097	0.7458
	QWPSO	0.810	0.7546	0.7423	0.7484

In summary, based on the two tests, we conclude that our proposed method of QWPSO offers an advantage when applied to typical MRI and CT medical image segmentation tasks, especially for segmenting complex indistinct tumor shapes. Compared with the existing methods of QPSO, SunCQPSO, CCQPSO SCQPSO, and IQPFLS, our approach offers improved performance in terms of operational efficiency. In addition, we reduced the running time to shorter and higher segmentation accuracy, both under manual observational inspection and in quantitative analysis using established evaluation parameters.

### Test 3: Compared With Existing Methods

To demonstrate the advantage of the proposed QWPSO method, test 3 implements a comparison between ten studies listed from (8, 68–77) in references whose publication years were from 2018 to 2021. Table 5 shows the original image, reference segmentation results, suggested QWPSO method, and ground truth in the 1st to 4th columns. The ground truth is obtained by an evaluation program. The detail is that we first input the original image into the evaluation program, and then input our segmented image. The program gave a standard segmentation result (red) based on the original image. Our segmentation result was blue, red, and blue overlap, indicating a good segmentation result. As seen in Table 5, despite the great challenge of these images due to the low contrast and high-intensity inhomogeneities, the QWPSO segmentation results are pretty consistent with the ground truth, and it successfully recovers the contours of the tumor substructures, especially in the region with bottle-neck. Although, for example, the green circle regions shown in the image of (8) illustrate the main differences between the segmentation result and the ground truth, their results lead to the fuzzy and missing of the bottle-neck parts. Still, our QWPSO segmentation results can enforce spatial consistency. Consequently, the contours of different reference images are well segmented by our QWPSO method.

**Table 5:** Comparison segmentation test.

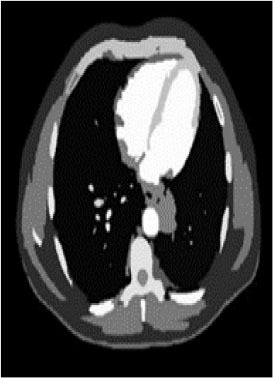 Original	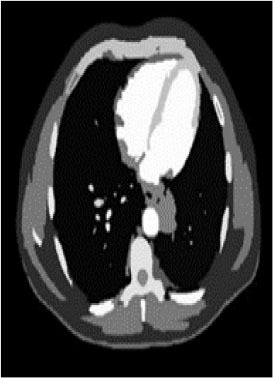 (68)	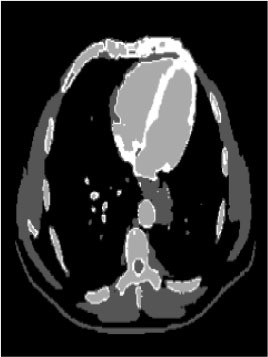 Ours	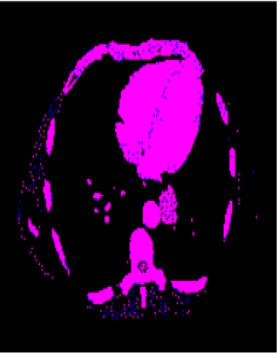 Ground Truth(G.T.)
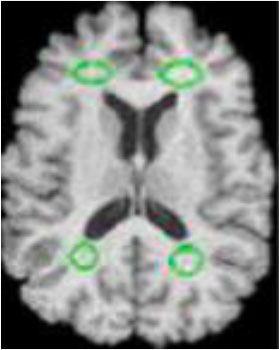 Original	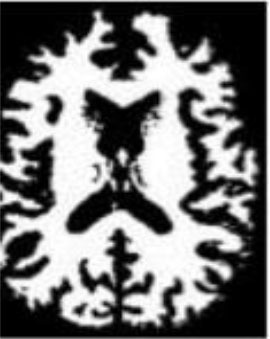 (8)	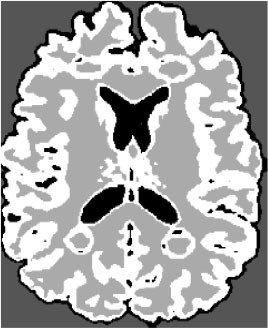 Ours	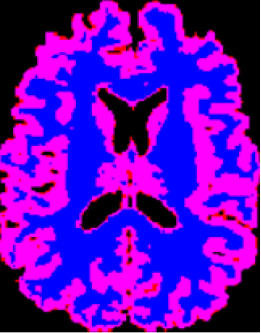 G.T.
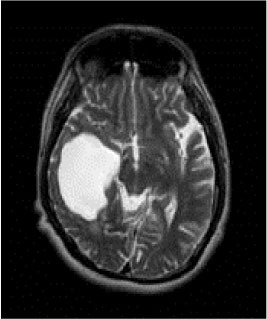 Original	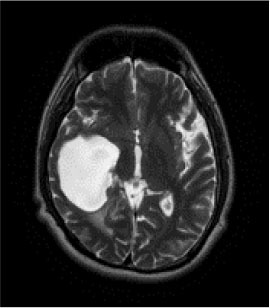 (69)	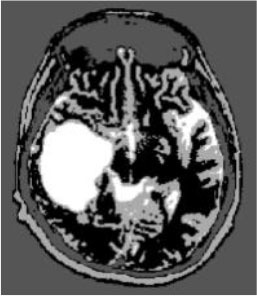 Ours	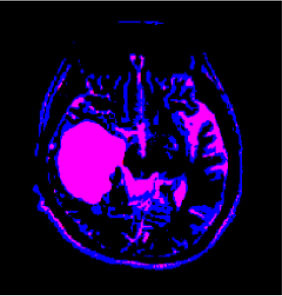 G.T.
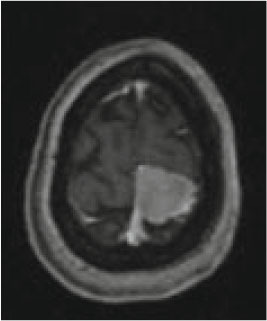 Original	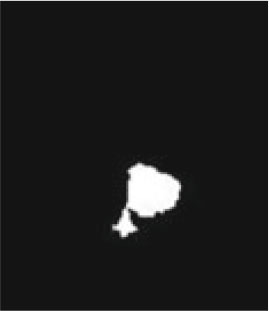 (70)	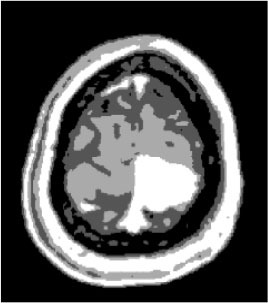 Ours	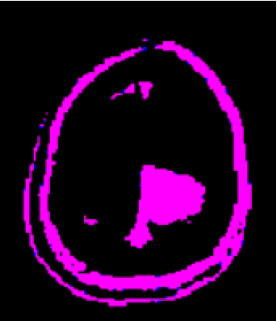 G.T.
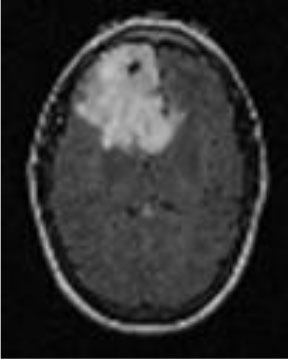 Original	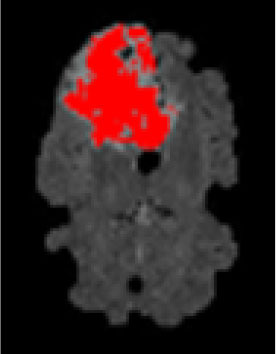 (71)	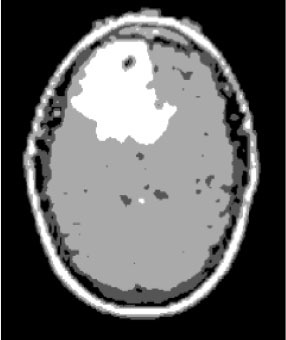 Ours	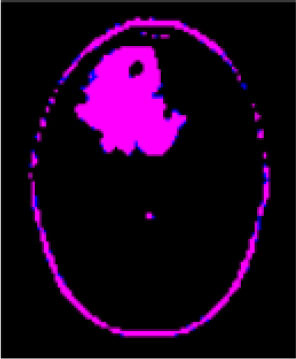 G.T.
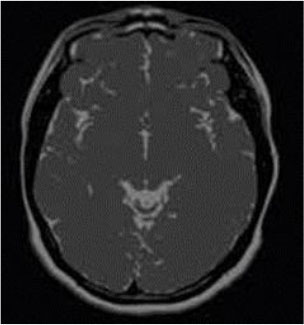 Original	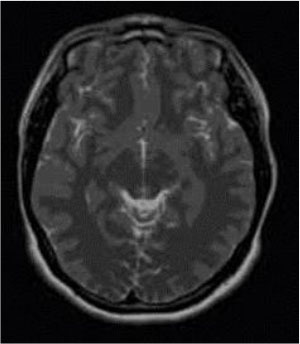 (72)	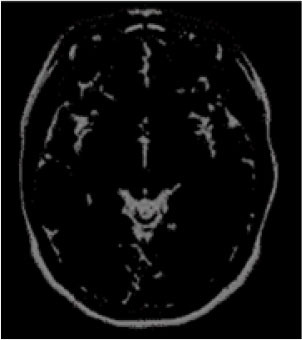 Ours	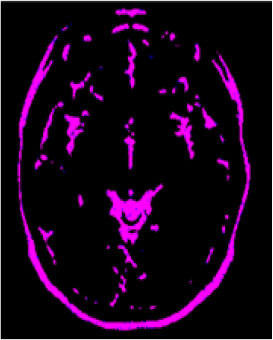 G.T.
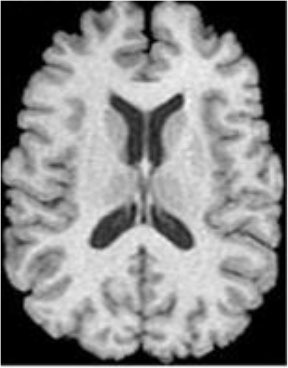 Original	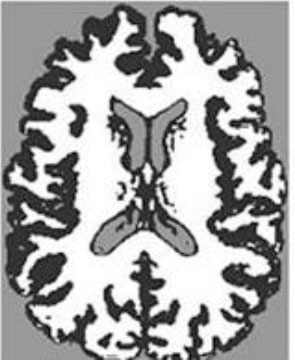 (73)	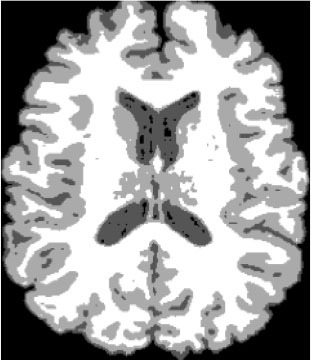 Ours	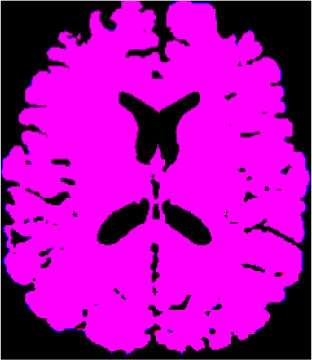 G.T.
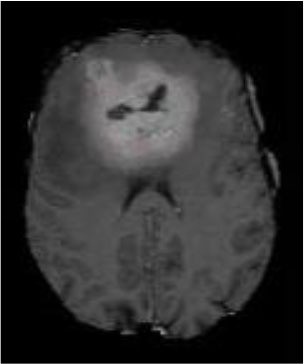 Original	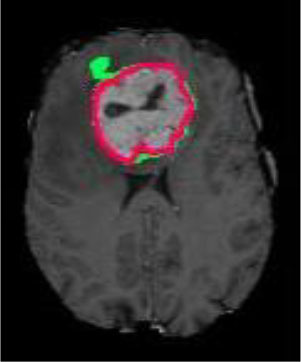 (74)	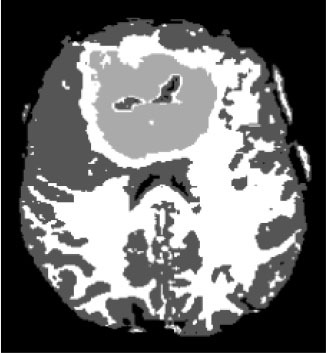 Ours	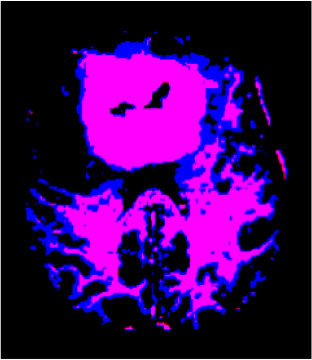 G.T.
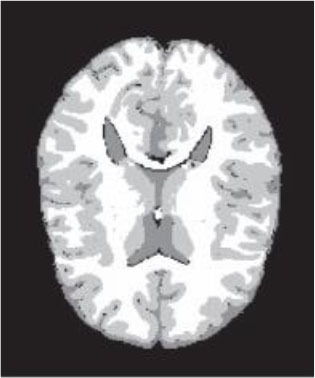 Original	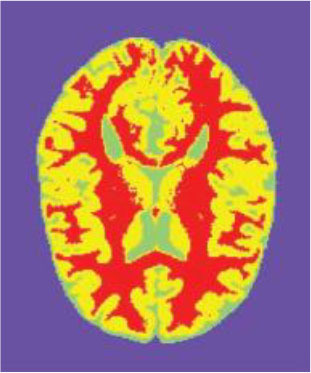 (75)	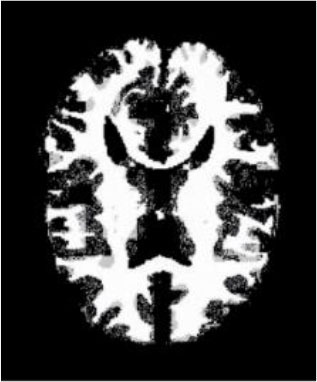 Ours	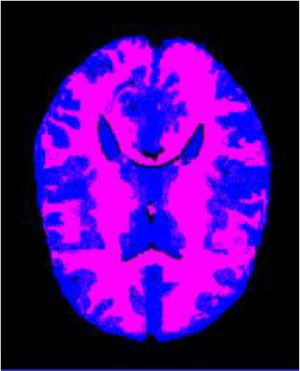 G.T.
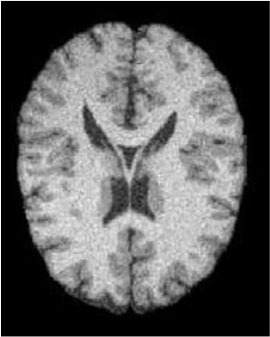 Original	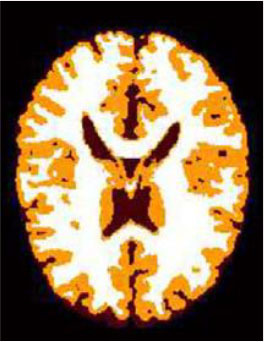 (76)	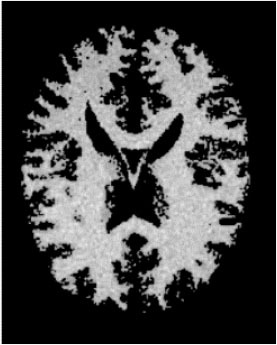 Ours	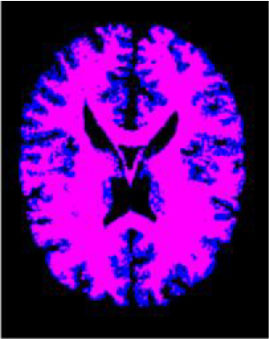 G.T.
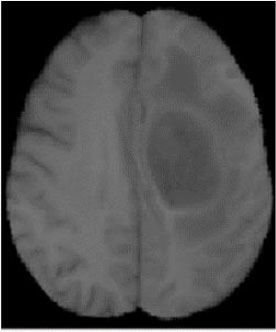 Original	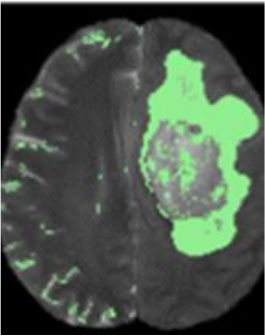 (77)	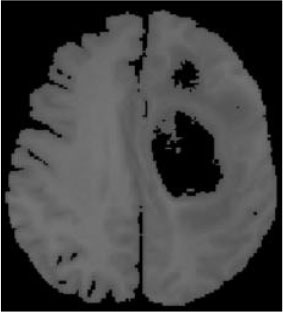 Ours	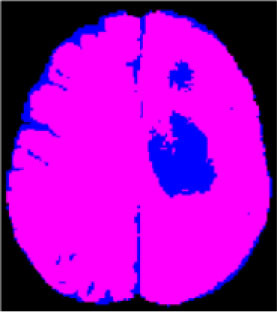 G.T.

**Figure 5:**
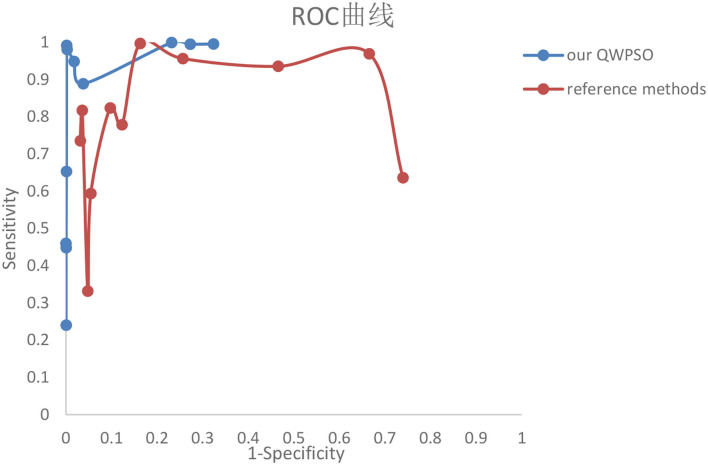
The receiver operating characteristic (ROC) curve of the proposed QWPSO and reference methods.

In addition to the P, R, and F parameters, the receiver operating curve (ROC) and Hausdorff evaluation parameters were added in this part in order to better compare the methods proposed and the experimental results of the reference studies. The ROC curve reflects the relationship between sensitivity and specificity, while the value of Hausdorff can measure the distance between proper subsets in a metric space. The smaller the Hausdorff value is, the higher the edge matching. ROC curve is shown in Figure 5, while Tables 6, 7 demonstrate the performance-evaluated parameters of segmentation Precision, Recall, F-measure, and Haudorff (H, the abbreviation for Haudorff in this study). All values of P, R, F, and H of the reference studies and our segmentation results are in each row.

**Table 6:** Evaluate parameters in test 3-part one.

	**Allioui et al. (68), Ours**	**Radha et al. (8), Ours**	**Mahesa et al. (69), Ours**	**Vijh et al. (70), Ours**	**Sharif et al. (71), Ours**
P	0.9176, 0.9785	0.8875, 0.9429	-, 0.9877	0.9800, 1.0000	0.994-0.998, 1.0000,
R	0.9248, 0.9541	-,0.5034	-,0.7088	-,0.9784	-,0.9265
F	0.9212,0.9661	-,0.6563	-,0.8253	-,0.9891	-, 0.9619
H	35.4683, 21.3073	24.5967, 18.1384	47.2017, 33.1813	93.1933, 10.9545	35.0571, 17.1172,

**Table 7:** Evaluate parameters in test 3-part two.

	**Khairuzzaman et al. (72), Ours**	**Ibungomacha Singh et al. (73), Ours**	**Hasan et al. (74), Ours**	**Guerrout et al. (75), Ours**	**Pham et al. (76), Ours**	**Ma et al. (77), Ours**
P	**0.7623–0.7911, 0.9789**,	**0.8650, 0.9993**	0.9220, 0.9656	0.855–0.991, 1.0000,	0.9126–0.9835, 1.0000	0.900, 0.9811
R	-,0.9479	**-,0.9920**	-,0.9544	-,0.5605	-,0.7218	0.850,0.8747
F	-, 0.9632	**-,0.9956**	-,0.9599	-, 0.7184	-,0.8384	0.870,0.9248
H	70.3847,17.5784	**22.0907, 3**	44.2945,28.7054	15.9374,14.6969	24.7790,10.1980	50.2905,17.8045

Table 6 demonstrates the reference studies (8, 68–71) published from 2021 to 2020. These studies used newly proposed methods within two years of the methods' publication in higher-level international journals from 2018 to 2019. Table 7 includes references (72–77) published from 2019 to 2018. In Table 6, for example, the first row P of Allioui et al. (68) is 0.9176, and the value of 0.9785 is the P results of our proposed QWPSO method. The corresponding segmentation results in rows P, R, and F show that our proposed methods are higher than their reference studies.

The first row in Table 7 shows that the mean values of P compared to (72) are from 0.7623 to 0.7911. Our value of 0.9789 is the average P. As for the last row of Haudorff, our H values are all less than the compared values, with the least one being three. Despite this, the H value compared with (73) is 22.0907, and the corresponding Ground Truth (GT) image in Table 5 is in pink without blue, indicating that the blue is completely overlapped. In addition, the segmented image and the standard segmentation image are almost identical and cannot be distinguished by the naked eye. Thus, regardless of whehter the studies were published in the periods 2018–2019 or 2020–2021, the segmentation results of our proposed QWPSO method are all higher than theirs. This further illustrates that the segmented images with prominent bottle-neck regions have even better performance, i.e., reference images of (70, 71, 75, 76), of which our evaluated parameter P are all 1.0000.

The ROC curve, also known as the “subject operating characteristic curve” or sensitivity curve, is mainly used for the prediction accuracy from X to Y. The ROC curve reflects the relationship between sensitivity and specificity. The X-axis is 1–specificity, also known as false-positive rate. The closer the X-axis is to zero, the higher the accuracy. The Y-axis is called sensitivity, also known as true positive rate (sensitivity). The higher the Y-axis, the better the accuracy. According to the position of the Curve, the whole graph is divided into two parts. The Area under the Curve (AUC) indicates the accuracy of prediction. The higher the AUC value is, the higher the accuracy of prediction. The closer the curve is to the top left corner. Hence, the smaller the X, the larger the Y, and the higher the prediction accuracy. The ROC curve of the segmentation results of our proposed method and reference studies are shown in Figure 5.

In comparing the two curves of our proposed QWPSO method and the reference methods in Figure 5, we can state that no matter the AUC, our proposed QWPSO method is better than the reference studies. Therefore, we conclude that our proposed QWPSO method has a higher segmentation accuracy than the reference methods.

## Discussion

The current related works with the proposed QWPSO are the PSO, QPSO, and their improving algorithms, such as SunCQPSO, CCQPSO SCQPSO, and IQPFLS. The limitations of all these related works are that they solve the general shape of the image segmentation problem rather than specifically for the special curved 'bottle-neck' shape of the image segmentation problem. However, in terms of technical improvement, there exists an interesting evolutionary relationship between the PSO, QPSO, and our QWPSO segmentation algorithm. While our proposed QWPSO algorithm is based on the existing QPSO technique, the QPSO approach is an improved version of the PSO algorithm. It is perhaps helpful to consider these algorithms' unique properties and functions. Particles are dynamically represented in the PSO approach. Particles adjust their speed according to the flight experience of both individuals and groups. The PSO algorithm works well for some image segmentation tasks but works less for others, particularly those involving complex and indistinct object shapes. The QPSO algorithm represents the latest intelligent optimization algorithm. Each particle moves well according to quantum behavior based on a Delta potential where they are centered during the various iteration steps. In this way, the QPSO algorithm can usefully enhance the population diversity and has a more robust global searching ability than the earlier PSO algorithm. Furthermore, this also means that the QPSO algorithm is better for more complex object shapes than in segmentation tasks. However, in the case of our QWPSO algorithm, all nodes are additionally considered to potentially have existing similar nodes which are more likely to be connected by a wormhole. This characteristic of the QWPSO algorithm provides a unique, advantageous, and powerful global searching ability for defining complex and unique object contour shapes in challenging image segmentation tasks. This unique ability to connect long-distance particles is a significant contribution of our QWPSO algorithm.

The above experimental results indicate that the QWPSO algorithm has good application for complex and specialized object contour segmentation, particularly for object regions typically encountered in medical tumor images that possess 'dual tails' and 'bottle-neck' feature shapes. For example, in Table 2, for the segmentation of MRI brain images, we present evaluation parameters that include Time, P, R, and F. We obtained the best values from the proposed QWPSO algorithm, namely, 0.801, 1.000, 1.000, and 0.9986 for Time, P, R, and F, respectively. Furthermore, the average running time of the QWPSO algorithm is 0.8743 s, which compares favorably with 0.9404 s for the QPSO algorithm, giving a decrease of 0.0961/s or ~12% reduction in average running time. Together these results indicate that our QWPSO algorithm has high efficiency in segmenting complex and specially shaped objects. As for the evaluation parameters of P, R, and F, our QWPSO algorithm attains optimal values of almost 1 for all. A parameter *p*-value of 0.9995 was obtained. While parameter R was slightly better for the QPSO algorithm, parameter F, which is the product of P and R, was better in all cases for our proposed QWPSO approach. It indicates that the accuracy of our proposed QWPSO algorithm is better than the QPSO algorithm for MRI brain image segmentation. Table 4 presents results obtained for the segmentation of CT brain images. All parameters, Time, P, R, and F, indicate improved performance over the improved QPSO algorithms, such as SunCQPSO, CCQPSO, and SCQPSO. Therefore, in general, the QWPSO algorithm offers greater adaptability to object region shape, together with better operational efficiency and segmentation accuracy over QPSO or improved QPSO algorithms for typically challenging MRI and CT brain image segmentation tasks. Furthermore, the evaluated parameters P, R, F, and H of the proposed QWPSO algorithm are shown in Tables 6, 7. All are better than the compared typical references within the last three years, especially achieving the highest value 1 for P among the images with distinct bottle-neck regions, such as images from references studies (70, 71, 75, 76). Lastly, the AUC area in the ROC curve of our proposed method in Figure 5 shows higher accuracy than that of all the reference studies.

## Conclusion

This study has presented a QWPSO algorithm for challenging image segmentation tasks. We applied wormhole-inspired theory to our method and put forward a hyperbolic wormhole path measure equation that seeds and links particles to improve the performance of the existing QPSO segmentation method. The QPSO method uses random positioning in the search space even if there are long distances between particles. Our QWPSO algorithm can cluster long-distance regions into groupings and has better adaptability than the existing QPSO algorithm and the current improved QPSO algorithms. Experimental results, both from MRI and CT, have demonstrated enhanced performance in segmenting rare brain tumors with tailing and bottle-neck regions. In addition, our QWPSO method improved operational efficiency and segmentation accuracy compared with current competing reference methods. Because there are many image segmentation that consists of similar curved targets, in the future, we are committed to extending the proposed QWPSO algorithm in this study to the segmentation of curved tumors in other organs' medical images, such as lung, liver, and/or spleen tumors. In addition to medical images, the proposed QWPSO algorithm should also be extended to other research areas of curving or bending target image segmentations.

## Data Availability Statement

Publicly available datasets were analyzed in this study. This data can be found here: https://mp.weixin.qq.com/s/d1H2j5vjrVo-RHRAVI8ygQ; https://www.med.upenn.edu/cbica/brats2020/data.html; https://www.med.upenn.edu/cbica/brats2019/data.html; https://www.med.upenn.edu/sbia/brats2018/data.html.

## Author Contributions

TZ designed the method, conducted the experiments, and wrote the manuscript. JZ helped to improve the results and modified the manuscript. TX handled this project. MR revised the manuscript and figures and enhanced their overall quality. All authors read this revised manuscript and gave final approval for the final submission.

## Funding

This research was supported by: (1) 2021–2023 National Natural Science Foundation of China under Grand (Youth) No. 52001039. (2) 2022–2025 National Natural Science Foundation of China under Grant No. 52171310. (3) 2020–2022 Shandong Natural Science Foundation Funding in China No. ZR2019LZH005. (4) 2022–2023 Research fund from Science and Technology on Underwater Vehicle Technology Laboratory under Grant 2021JCJQ-SYSJJ-LB06903.

## Conflict of Interest

The authors declare that the research was conducted in the absence of any commercial or financial relationships that could be construed as a potential conflict of interest.

## Publisher's Note

All claims expressed in this article are solely those of the authors and do not necessarily represent those of their affiliated organizations, or those of the publisher, the editors and the reviewers. Any product that may be evaluated in this article, or claim that may be made by its manufacturer, is not guaranteed or endorsed by the publisher.
