# Supplementary material for: A Brain Tumor Image Segmentation Method Based on Quantum Entanglement and Wormhole Behaved Particle Swarm Optimization
Source: Front Med (Lausanne). 2022 May 10;9:794126. doi: 10.3389/fmed.2022.794126 (PMC9127532; doi:10.3389/fmed.2022.794126)
Supplement: Supplementary file 1 [file Table_1.pdf]

Appendix

Symbol	Equation	Explanation
c	$\mathrm{d}s^2 =- c^2\mathrm{d}t^2 + \frac{\mathrm{d}r^2}{1-\frac{b_0^2}{r^2}} + r^2(\mathrm{d}\theta^2 + \sin^2\theta\mathrm{d}\varphi^2)$ (1)	the speed of light
θ	$\mathrm{d}s^2 =- c^2\mathrm{d}t^2 + \frac{\mathrm{d}r^2}{1-\frac{b_0^2}{r^2}} + r^2(\mathrm{d}\theta^2 + \sin^2\theta\mathrm{d}\varphi^2)$ (1)	the zenith angle between positive z-axis
r	$ds^2 =- c^2dt^2 + \frac{dr^2}{1-\frac{b_0^2}{r^2}} + r^2(d\theta^2 + sin^2\theta d\varphi^2)$ (1)	the radius of the throat part of the wormhole.
	$\mathrm{d}s^2 = \frac{\mathrm{d}r^2}{1-\frac{b_0^2}{r^2}} + r^2\mathrm{d}\varphi^2$ (2)	
	$z(r) =\pm b_0 \ln\left[\frac{r}{b_0} + \sqrt{\left(\frac{r}{b_0}\right)^2 - 1}\right]$ (3)	
φ	$ds^2 =- c^2dt^2 + \frac{dr^2}{1-\frac{b_0^2}{r^2}} + r^2(d\theta^2 + sin^2\theta d\varphi^2)$ (1)	the azimuth angle between positive X-axis in spherical coordinate system
	$\mathrm{d}s^2 = \frac{\mathrm{d}r^2}{1-\frac{b_0^2}{r^2}} + r^2\mathrm{d}\varphi^2$ (2)	
b_0	$ds^2 =- c^2dt^2 + \frac{dr^2}{1-\frac{b_0^2}{r^2}} + r^2(d\theta^2 + sin^2\theta d\varphi^2)$ (1)	b_0=2GM, where M is the object's mass, G is the universal gravitational constant
	$\mathrm{d}s^2 = \frac{\mathrm{d}r^2}{1-\frac{b_0^2}{r^2}} + r^2\mathrm{d}\varphi^2$ (2)	
	$z(r) =\pm b_0 \ln\left[\frac{r}{b_0} + \sqrt{\left(\frac{r}{b_0}\right)^2 - 1}\right]$ (3)	

	$z(r) = \pm b_0 \ln(a)$ (4)	
$z(r)$	$z(r) = \pm b_0 \ln(a)$ (4)	the spatial shape of the entire hyperboloid obtained by rotating numerous radius lines
r, r'	$x(r) = r + r' + \left(\frac{2}{\zeta}\right)\ln\left(\frac{\Delta\theta}{2}\right)$ (5)<br>$x = r + r' + \left(\frac{2}{\zeta}\right)\ln\left(\frac{\Delta\theta}{2}\right)$ (13)	Two particle positions of radiuses at a distance from the disc center
ζ	$x(r) = r + r' + \left(\frac{2}{\zeta}\right)\ln\left(\frac{\Delta\theta}{2}\right)$ (5)<br>$x = r + r' + \left(\frac{2}{\zeta}\right)\ln\left(\frac{\Delta\theta}{2}\right)$ (13)<br>$X_{iw} = P_{id} \pm \left(\frac{2}{\zeta}\right)\ln\left(\frac{\Delta\theta}{2}\right)$ (15)<br>$\mathrm{x(t+1)} = P(t) - \left(\frac{2}{\zeta}\right) \cdot \lvert \mathrm{Mbest} - \mathrm{x(t)} \rvert \cdot \ln\left(\frac{\Delta\theta}{2}\right)$ (16)<br>$\mathrm{x(t+1)} = P(t) + (2/\zeta) \cdot \lvert \mathrm{Mbest} - \mathrm{x(t)} \rvert \cdot \ln\left(\frac{\Delta\theta}{2}\right)$ （17）	the distance coefficient
$\Delta\theta$	$x(r) = r + r' + \left(\frac{2}{\zeta}\right)\ln\left(\frac{\Delta\theta}{2}\right)$ (5)<br>$x = r + r' + \left(\frac{2}{\zeta}\right)\ln\left(\frac{\Delta\theta}{2}\right)$ (13)<br>$X_{iw} = P_{id} \pm \left(\frac{2}{\zeta}\right)\ln\left(\frac{\Delta\theta}{2}\right)$ (15)<br>$\mathrm{x(t+1)} = P(t) - \left(\frac{2}{\zeta}\right) \cdot \lvert \mathrm{Mbest} - \mathrm{x(t)} \rvert \cdot \ln\left(\frac{\Delta\theta}{2}\right)$ (16)	The range of $\Delta\theta$ is: $0 < \Delta\theta < 57.32$

	$x(t+1) = P(t) + (2/\zeta) \cdot \lvert Mbest - x(t) \rvert \cdot \ln\left(\frac{\Delta\theta}{2}\right)$ （17）	
$Q(y_{id})$	$Q(y_{id}) = \lvert\psi(y_{id})\rvert^2 = \frac{1}{L}e^{\frac{-2\lvert y_{id}\rvert}{L}}$ (6)	the location of a particle probabilistically
$\psi(y_{id})$	$Q(y_{id}) = \lvert\psi(y_{id})\rvert^2 = \frac{1}{L}e^{\frac{-2\lvert y_{id}\rvert}{L}}$ (6)	the spin field operator
L	$Q(y_{id}) = \lvert\psi(y_{id})\rvert^2 = \frac{1}{L}e^{\frac{-2\lvert y_{id}\rvert}{L}}$ (6)	the characteristic length of the potential well
s	$s = \frac{1}{L}rand(0,1) = \frac{1}{L}u, and\ u = rand(0,1)$ (7)	is a lucky number within the range of $(0, 1/L)$
u	$s = \frac{1}{L}rand(0,1) = \frac{1}{L}u, and\ u = rand(0,1)$ (7)	the random value between 0 and 1 that represents the arbitrary distance between particles in the quantum potential well
P	$P = \frac{(\varphi_1 \times P_{id} + \varphi_2 \times P_{gd})}{\varphi_1 + \varphi_2}$ (9)	the particles coordinates
	$x(t+1) = P - \alpha \cdot \lvert Mbest - x(t) \rvert \cdot \ln\left(\frac{1}{\mu}\right)$ （11）	
	$x(t+1) = P + \alpha \cdot \lvert Mbest - x(t) \rvert \cdot \ln\left(\frac{1}{\mu}\right)$ （12）	
	$x(t+1) = P(t) - \left(\frac{2}{\zeta}\right) \cdot \lvert Mbest - x(t) \rvert \cdot \ln\left(\frac{\Delta\theta}{2}\right)$ (16)	
	$x(t+1) = P(t) + (2/\zeta) \cdot \lvert Mbest - x(t) \rvert \cdot \ln\left(\frac{\Delta\theta}{2}\right)$ （17）	
φ_1	$P = \frac{(\varphi_1 \times P_{id} + \varphi_2 \times P_{gd})}{\varphi_1 + \varphi_2}$ (9)	$\varphi_1 = rand(0,1)$

φ_2	$P = \frac{(\varphi_1 \times P_{id} + \varphi_2 \times P_{gd})}{\varphi_1 + \varphi_2}$ （9）	$\varphi_2 = \mathrm{rand}(0,1)$
P_{id}	$P = \frac{(\varphi_1 \times P_{id} + \varphi_2 \times P_{gd})}{\varphi_1 + \varphi_2}$ (9)<br>$X_{iw} = P_{id} \pm \left(\frac{2}{\zeta}\right) \ln\left(\frac{\Delta\theta}{2}\right)$ (15)	the best position of the particle
P_{gd}	$P = \frac{(\varphi_1 \times P_{id} + \varphi_2 \times P_{gd})}{\varphi_1 + \varphi_2}$ （9）	the global best position of the population
Mbest	$\mathrm{Mbest} = \frac{\sum_{i=1}^{M} P(t)}{M}$<br>$x(t+1) = P - \alpha \cdot \lvert \mathrm{Mbest} - x(t) \rvert \cdot \ln\left(\frac{1}{\mu}\right)$ （11）<br>$x(t+1) = P + \alpha \cdot \lvert \mathrm{Mbest} - x(t) \rvert \cdot \ln\left(\frac{1}{\mu}\right)$ （12）<br>$x(t+1) = P(t) - \left(\frac{2}{\zeta}\right) \cdot \lvert \mathrm{Mbest} - x(t) \rvert \cdot \ln\left(\frac{\Delta\theta}{2}\right)$ (16)<br>$x(t+1) = P(t) + (2/\zeta) \cdot \lvert \mathrm{Mbest} - x(t) \rvert \cdot \ln\left(\frac{\Delta\theta}{2}\right)$ （17）	the mean best position
M	$\mathrm{Mbest} = \frac{\sum_{i=1}^{M} P(t)}{M}$ （10）	represents the number of particles
$P(t)$	$\mathrm{Mbest} = \frac{\sum_{i=1}^{M} P(t)}{M}$ （10）	represents the position of the particle P_{id} at time t
x	$x(t+1) = P - \alpha \cdot \lvert \mathrm{Mbest} - x(t) \rvert \cdot \ln\left(\frac{1}{\mu}\right)$ （11）<br>$x(t+1) = P + \alpha \cdot \lvert \mathrm{Mbest} - x(t) \rvert \cdot \ln\left(\frac{1}{\mu}\right)$ （12）	the wormhole measure of hyperbolic path x

	$$x(t+1) = P(t) - \left(\frac{2}{\zeta}\right) \cdot \lvert Mbest - x(t) \rvert \cdot \ln\left(\frac{\Delta\theta}{2}\right) \quad (16)$$ $$x(t+1) = P(t) + (2/\zeta) \cdot \lvert Mbest - x(t) \rvert \cdot \ln\left(\frac{\Delta\theta}{2}\right) \quad (17)$$	
α	$$x(t+1) = P - \alpha \cdot \lvert Mbest - x(t) \rvert \cdot \ln\left(\frac{1}{\mu}\right) \quad (11)$$ $$x(t+1) = P + \alpha \cdot \lvert Mbest - x(t) \rvert \cdot \ln\left(\frac{1}{\mu}\right) \quad (12)$$	the expansion coefficient of the speed in controlling convergence, and it represents the maximum number of iterations convergence
μ	$$x(t+1) = P - \alpha \cdot \lvert Mbest - x(t) \rvert \cdot \ln\left(\frac{1}{\mu}\right) \quad (11)$$ $$x(t+1) = P + \alpha \cdot \lvert Mbest - x(t) \rvert \cdot \ln\left(\frac{1}{\mu}\right) \quad (12)$$	random digital
x(t)	$$x(t+1) = P - \alpha \cdot \lvert Mbest - x(t) \rvert \cdot \ln\left(\frac{1}{\mu}\right) \quad (11)$$ $$x(t+1) = P + \alpha \cdot \lvert Mbest - x(t) \rvert \cdot \ln\left(\frac{1}{\mu}\right) \quad (12)$$ $$x(t+1) = P(t) - \left(\frac{2}{\zeta}\right) \cdot \lvert Mbest - x(t) \rvert \cdot \ln\left(\frac{\Delta\theta}{2}\right) \quad (16)$$ $$x(t+1) = P(t) + (2/\zeta) \cdot \lvert Mbest - x(t) \rvert \cdot \ln\left(\frac{\Delta\theta}{2}\right) \quad (17)$$	representing the next step for the iteration variable wormhole particle
$P(t)$	$$x(t+1) = P(t) - \left(\frac{2}{\zeta}\right) \cdot \lvert Mbest - x(t) \rvert \cdot \ln\left(\frac{\Delta\theta}{2}\right) \quad (16)$$	the position of the particle P_{id} at time t

	$x(t+1) = P(t) + (2/\zeta) \cdot \lvert \mathrm{Mbest} - x(t) \rvert \cdot \ln\left(\frac{\Delta\theta}{2}\right)$ （17）	
$\rho(r)$	$\rho(r) \approx e^{-\frac{\zeta r}{2}}$ (14)	the node probability distribution of the wormhole path measure
X_{iw}	$X_{iw} = P_{id} + \left(\frac{2}{\zeta}\right) \ln\left(\frac{\Delta\theta}{2}\right)$ (15)	the position of a particle in the wormhole path measure
Δf	$\Delta f = \lvert f_{ij} - f_{kl} \rvert \leq TH_f$ and $\Delta d = \sqrt{(i-k)^2 + (j-l)^2} \leq TH_o$ （18）<br>$\Delta f = \lvert f_{ij} - \bar{f} \rvert \leq TH_f$ and $\sqrt{(i-k)^2 + (j-l)^2} \leq TH_o$ （19）	the gray value difference of the two particle
f_{ij}	$\Delta f = \lvert f_{ij} - f_{kl} \rvert \leq TH_f$ and $\Delta d = \sqrt{(i-k)^2 + (j-l)^2} \leq TH_o$ （18）<br>$\Delta f = \lvert f_{ij} - \bar{f} \rvert \leq TH_f$ and $\sqrt{(i-k)^2 + (j-l)^2} \leq TH_o$ （19）	the gray seed particle value
f_{kl}	$\Delta f = \lvert f_{ij} - f_{kl} \rvert \leq TH_f$ and $\Delta d = \sqrt{(i-k)^2 + (j-l)^2} \leq TH_o$ （18）<br>$\Delta f = \lvert f_{ij} - \bar{f} \rvert \leq TH_f$ and $\sqrt{(i-k)^2 + (j-l)^2} \leq TH_o$ （19）	the other gray seed particle value
TH_f	$\Delta f = \lvert f_{ij} - f_{kl} \rvert \leq TH_f$ and $\Delta d = \sqrt{(i-k)^2 + (j-l)^2} \leq TH_o$ （18）<br>$\Delta f = \lvert f_{ij} - \bar{f} \rvert \leq TH_f$ and $\sqrt{(i-k)^2 + (j-l)^2} \leq TH_o$ （19）	the pixel gray value difference
TH_o	$\Delta f = \lvert f_{ij} - f_{kl} \rvert \leq TH_f$ and $\Delta d = \sqrt{(i-k)^2 + (j-l)^2} \leq TH_o$ （18）<br>$\Delta f = \lvert f_{ij} - \bar{f} \rvert \leq TH_f$ and $\sqrt{(i-k)^2 + (j-l)^2} \leq TH_o$ （19）	the threshold values of position variance

Δd	$\Delta f = \lvert f_{ij} - f_{kl} \rvert \leq TH_f$ and $\Delta d = \sqrt{(i-k)^2 + (j-l)^2} \leq TH_o$ （18）<br>$\Delta f = \lvert f_{ij} - \overline{f} \rvert \leq TH_f$ and $\Delta d = \sqrt{(i-k)^2 + (j-l)^2} \leq TH_o$（19）	the root mean square difference of the two particles position
$\overline{f}$	$\Delta f = \lvert f_{ij} - f_{kl} \rvert \leq TH_f$ and $\Delta d = \sqrt{(i-k)^2 + (j-l)^2} \leq TH_o$ （18）<br>$\Delta f = \lvert f_{ij} - \overline{f} \rvert \leq TH_f$ and $\Delta d = \sqrt{(i-k)^2 + (j-l)^2} \leq TH_o$（19）	the average gray value of the particles in the seed area

2
